# Effects of Cp*-Ligands in Titanocene-Based Thiosemicarbazone Complexes

**DOI:** 10.1021/acsomega.6c02122

**Published:** 2026-05-14

**Authors:** Kevin Schwitalla, Marc Schmidtmann

**Affiliations:** Institut Für Chemie, Carl von Ossietzky Universität Oldenburg, Oldenburg D-26111, Federal Republic of Germany

## Abstract

Titanocene-based complexes are among the most prominent organometallic anticancer drugs. Since the discovery of titanocene dichloride, numerous derivatives have been tested as potential chemotherapeutic agents. We previously reported on titanocene-based drug carriers by using substitution-labile triflato ligands to generate cationic complexes with cytotoxic thiosemicarbazones (TSCN). Building on this, we posed the question of whether the larger cyclopentadiene derivative, pentamethylcyclopentadiene (Cp*), might further stabilize the organometallic drug carriers or provide other benefits such as increased lipophilicity. In this work, we describe the synthesis of titanocene-based complexes bearing Cp* and TSCN ligands and discuss how the Cp* ligands affect the water solubility and stability. Our findings are supported by DFT calculations, which enable a rational approach for the design of novel organometallic drug carriers.

## Graphical abstract

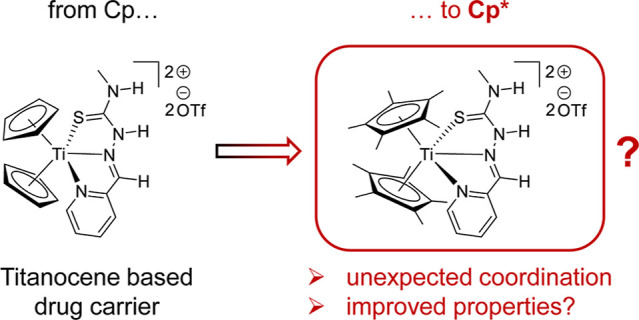

## Introduction

Drug delivery is one of the most important aspects of medicinal chemistry as it determines the specificity and efficiency of pharmaceuticals.^1,2^ Since many drugs are either not specific or suited to biological environments inside the body, drug delivery systems (DDSs) have been developed to promote and improve drug delivery.^3,4^ Besides nanoparticles^5,6^ and proteinogenic DDSs,^7,8^ inorganic and organometallic complexes have been used as efficient drug carriers, known as “metallodrugs”.^9–11^ The most prominent example is certainly the anticancer drug cisplatin, which has been widely used since its discovery.^12,13^ However, because cisplatin and other platinum-based anticancer drugs are associated with severe side-effects during therapy, there has been a longstanding desire to create drugs that eventually replace these complexes.^14^ One of the most well-known example for an organometallic metallodrug is titanocene dichloride,^15^ which was extensively studied as an alternative to cisplatin. However, due to insufficient activity during clinical trials,^16^ further research on titanocene dichloride was discontinued. Since then, many other titanium-based metallodrugs have been developed, such as budotitan,^17^ amine-phenolato-,^18^ salen-,^19,20^ salalen,^21^ and more recently deferasirox-based complexes.^22^

Recently, we developed titanocene-based drug carriers that enhance the water solubility of thiosemicarbazones (TSCNs) by generating cationic complexes through the exchange reaction of the substitution-labile triflato ligands with multidentate ligands.^23^ Such TSCNs and related metal complexes exhibit several biological activities, such as antimicrobial,^24–26^ antiviral,^27–29^ and anticancer properties.^30–34^ The bioactive but usually water-insoluble TSCNs can, by metal complexation, be used more effectively in aqueous environments with a higher activity in comparison to the pure drug. Despite this, there are still drawbacks to this approach. While the titanium complexes are stable in water for several hours, their stability could be further improved, particularly in aqueous buffers, where we observe fast hydrolysis of the complexes, which could limit the effectiveness of the drug carrier in the human body. One potential strategy to overcome this issue is to alter the cyclopentadienyl (Cp) ligands since the stability of metallocenes can be controlled by choosing optimal types of Cp ligands.^35^ The larger derivative of Cp, namely, pentamethylcyclopentadienyl (Cp*), is known to stabilize complexes more efficiently than Cp ligands.^35,36^ Therefore, we aimed to use Cp* ligands to stabilize the titanocene-based complexes while also improving the lipophilicity of the compounds caused by the numerous methyl groups of the Cp* ligand. In this work, we demonstrate the synthesis of titanocene-based complexes with Cp* ligands and create cations with TSCN ligands via the displacement of substitution-labile triflato ligands. We discuss the effects caused by the Cp* ligands on the coordination mode of the complexes as well as the stability in aqueous media and validate our findings with quantum chemical calculations.

## Results and Discussion

### Synthesis and Coordination Chemistry

For the synthesis of monocationic complexes, we previously reported the reaction of titanocene bis­(trimethylsilyl)­acetylene with thiosemicarbazones and subsequent oxidation with ferrocenium salts.^23^ Similarly, the reaction of the di­(pentamethylcyclopentadienyl)­titanium bis­(trimethylsilyl)­acetylene complex **Ti1** with *N*-heterocyclic thiosemicarbazone **a** leads to the formation of the respective Ti­(III) complex **Ti1a** (Scheme 1). The redox reaction is triggered by the masked titanocene­(II) species, which deprotonates the acidic N^β^-proton of the ligands by releasing bis­(trimethylsilyl)­acetylene (BTMSA) and dihydrogen.^36^ In this case, elevated temperatures of 60 °C are required due to the higher stability of the Cp* bis­(trimethylsilyl)­acetylene derivative.^36^ The κ^2^ *N* ^β^ *,S* coordination mode of complex **Ti1a** was determined by single-crystal X-ray crystallography (Figure 1) and is consistent with the reported Cp-derivatives.^23^ The Ti­(III) character of **Ti1a** was measured with EPR spectroscopy (**ESI**, Figure S1). **Ti1a** was subsequently oxidized with ferrocenium triflate to obtain the cationic Ti­(IV) complex **Ti2a** with a triflate anion (Scheme 1). The coordination mode of **Ti2a** was identified by single-crystal X-ray diffraction (Figure 1), again revealing a κ^2^ *N* ^β^,*S* coordination mode in the solid state, which is contrary to the Cp-derivatives, where a κ^3^ *N*,*N*,*S* coordination was found after oxidation of the respective Ti­(III) complex.^23^

**Scheme 1:**
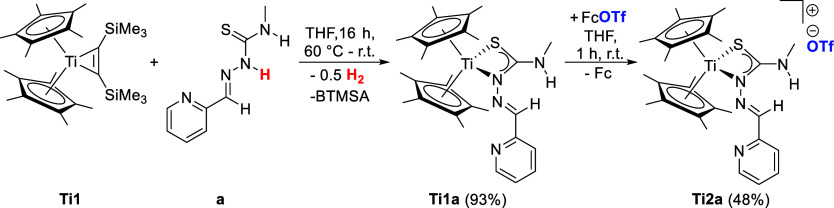
Reaction of Di­(pentamethylcyclopentadienyl)­titanium bis­(trimethylsilyl)­acetylene **Ti1** with TSCN **a** to Obtain Ti­(III) Thiosemicarbazonato Complexes **Ti1a** ^a^

a Reaction of **Ti1a** with ferrocenium triflate to obtain the cationic complex **Ti2a** with a triflate anion.

**Figure 1:**
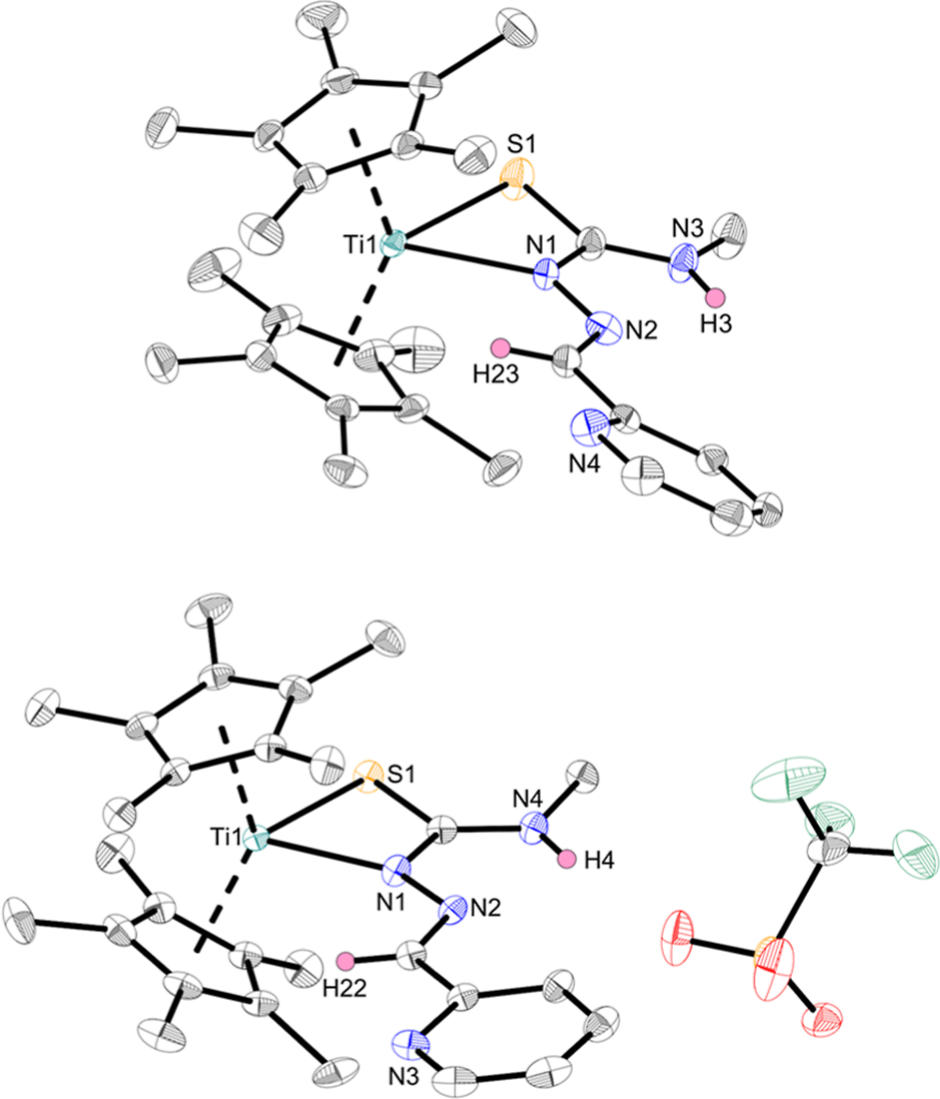
Crystal structures of complex **Ti1a** (top) and **Ti2a** (bottom). Displacement ellipsoids are drawn at the 50% probability level. Redundant H atoms have been omitted for clarity.

The divergent coordination mode of **Ti2a** is most likely due to electronic saturation of the metal, which is caused by the increased electron-donating effect and the increased steric hindrance of the Cp* ligand in contrast to Cp.^36^ To further investigate this, we synthesized the corresponding dicationic complex. Dicationic complexes are considered to have a higher water solubility and cytotoxic activity, which was previously described for the Cp derivatives.^23^ Cationic complexes with triflate anions can be generated by displacing substitution-labile triflato ligands with multidentate, neutral ligands.^37^

By reacting the di­(pentamethylcyclopentadienyl)­titanium­(IV)­triflato complex **Ti3** with TSCN **a** (Scheme 2), the dicationic complex **Ti3a** is obtained by displacing the two coordinating triflato ligands with the thiosemicarbazone. Surprisingly, instead of forming the reported κ^3^ *N*,*N* ^α^,*S* coordination mode for the respective Cp derivatives,^23^ the crystal structure of **Ti3a** revealed a κ^2^ *N* ^β^,*S* coordination mode (Figure 2), while the N–H proton of the TSCN ligand was transferred to the pyridyl moiety, forming a pyridinium ion. Because of this surprising difference in coordination modes, we calculated both forms using DFT calculations. The optimized geometries were calculated at the B3LYP/Def2-TZVP level of theory using the SMD solvation model with THF as the solvent.^38^ They revealed an approximate 54 kJ/mol difference in free energy between both forms, with the given κ^2^ *N* ^β^,*S* coordination being thermodynamically preferred to the κ^3^ *N*,*N* ^α^,*S* coordination mode (**Ti3a** ^**2+**^ **NNS**, **ESI** Table S3). This significant difference in free energies can be explained by both the steric and electronic effects of the Cp* ligands.^36^ On one hand, the higher steric demand of Cp* hinders the formation of the more demanding κ^3^ *N*,*N* ^α^,*S* coordination and favors the κ^2^ *N* ^β^,*S* coordination. On the other hand, due to the increased electron donation of the Cp* ligand in comparison with a Cp ligand, the formal 16 electron complex of the κ^2^ *N* ^β^,*S* coordination mode is stabilized, while the formal 18 electron κ^3^ *N*,*N* ^α^,*S* complex does not benefit from the additional electron density as it is electronically saturated.^39^

**Scheme 2:**
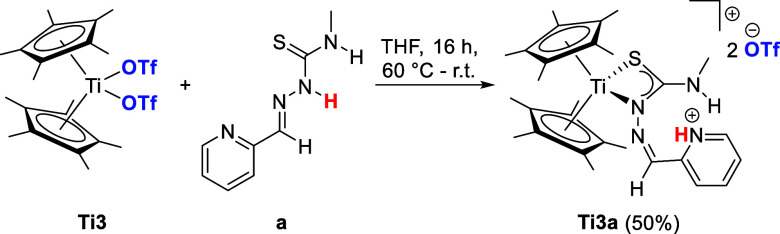
Reaction of Di­(pentamethylcyclopentadienyl)­titanium Bistriflate **Ti3** with TSCN **a** to Obtain the Dicationic Ti­(IV) Thiosemicarbazonato Complex **Ti3a** with Triflate Anions

**Figure 2:**
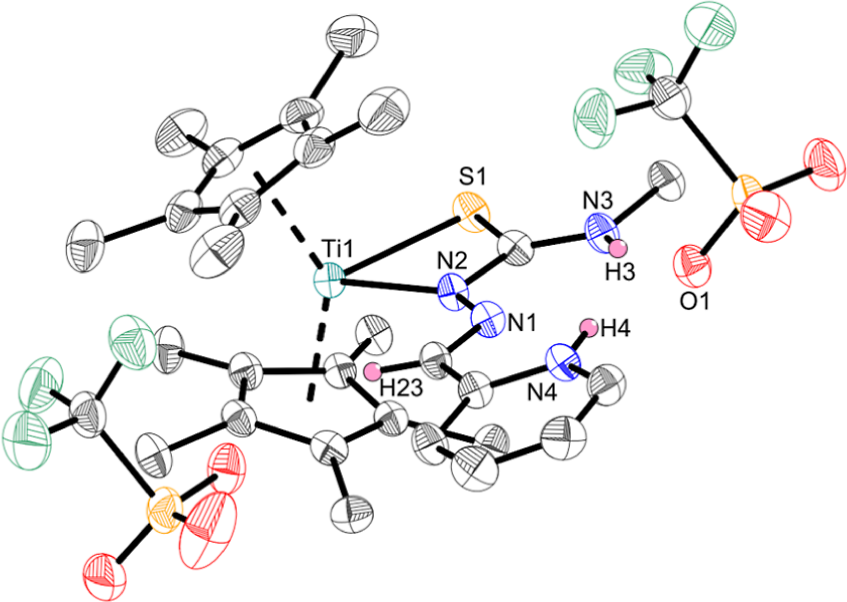
Crystal structure of complex **Ti3a**. Displacement ellipsoids are drawn at the 50% probability level. Redundant H atoms have been omitted for clarity.

The infrared spectra support the differences in coordination modes as the spectrum of **Ti3a** shows additional bands (e.g., 2850–2400 (br), 1621, 1557, 1554, 1539 cm^–1^) that are not visible in the spectra of **Ti1a** and **Ti2a** and could indicate the presence of the pyridinium ion. Due to the similar NMR chemical shifts in comparison with **Ti2a**, the pyridinium ion form of **Ti3a** may not necessarily be present in solution. To understand the potential mechanism toward the formation of the pyridinium ion in THF solution, we have modeled the intramolecular proton transfer reaction using DFT calculations (Scheme 3). The optimized geometries were calculated using the above applied level of theory.^38^ The mechanism involves a κ^2^ *N* ^*α*^ *,S* coordination mode **A** that enables the 1,5 H-shift for the protolysis of the pyridine moiety. We propose that this mode is initially formed by the coordination of the neutral TSCN ligand. This reaction has a very low activation energy (**TS1**) of +9 kJ/mol relative to the starting complex **A** and leads to intermediate **B** with a κ^2^ *N* ^*α*^ *,S* coordination mode and a protonated pyridine, with a slightly lower free energy of −2 kJ/mol in comparison with **A**. The transition state (**TS1**) shows a direct transfer of the proton between both nitrogen atoms with N–H bond lengths of 1.27 Å (**ESI**, Table S2). Then, a switch of the coordination mode (**TS2**) occurs to alternate from the κ^2^ *N* ^α^,*S* coordination in **B** to the κ^2^ *N* ^β^,*S* coordination of the final product **Ti3a** ^**2+**^. The energy barrier of this reaction of +111 kJ/mol is relatively high but accessible at room temperature and more so at higher temperatures as this reaction proceeds at 60 °C. The coordination switch occurs by dissociation of the Ti–N bond of **B**, leading to a κ^1^ *S* coordination in the transition state (**TS2**) and subsequent coordination of the *N* ^β^ atom, resulting in the formation of **Ti3a** ^**2+**^. While other conformers of **TS2** are imaginable, the transition state was calculated free of restrictions and is the energetically favored state. As the reaction was performed at elevated temperatures for 16 h, this intramolecular reaction mechanism is reasonable and could be described as an equilibrium. Alternatively, intermolecular mechanisms are also imaginable, either mediated by a triflate anion or via a second complex.

**Scheme 3:**
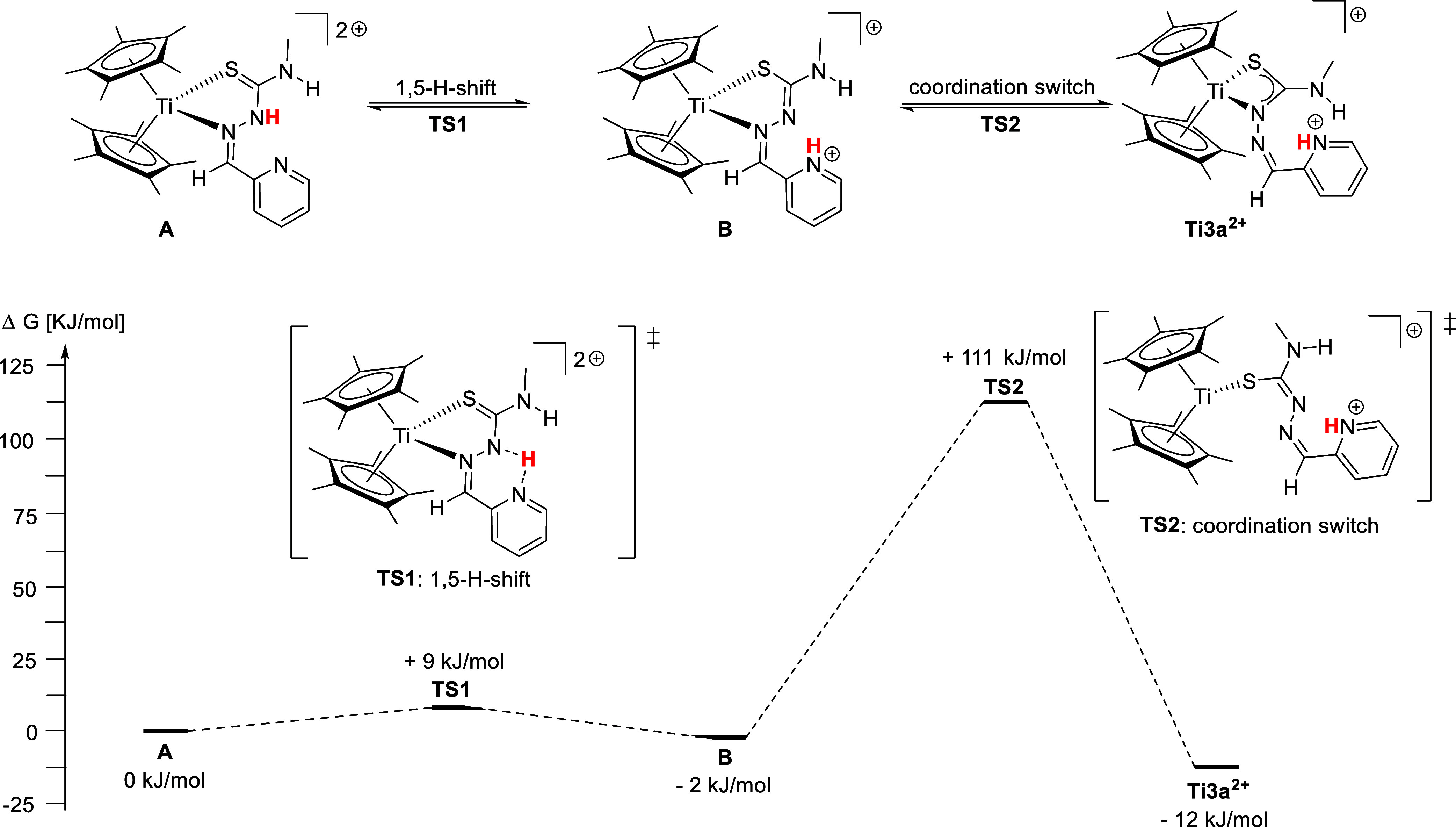
Top: Intramolecular Proton Transfer Reaction and Coordination Switch for the Formation of **Ti3a** ^a^

a Bottom: free energy profile of the reaction mechanism.

### Water Solubility and Stability of the Complexes

In contrast to the Cp derivatives of the TSCN complexes, which are moderately soluble and stable in aqueous solutions,^23^ the Cp* derivatives show poor solubility and stability in water. The lessened solubility of the compounds in aqueous media is most likely due to the increased lipophilicity that is caused by the additional methyl groups of the Cp* ligand. The poor aqueous stability is demonstrated by ^1^H NMR experiments in D_2_O, which resulted in instant hydrolysis of the complexes, forming [Cp*_2_Ti­(D_2_O)_2_]­(OTf)_2_, among other products (e.g., [Cp_2_Ti­(D_2_O)-μO-Cp_2_Ti­(D_2_O)]­(OTf)_2_), as hydrolysis products for **Ti2a** and **Ti3a** (**ESI**, Figures S7 and S8). The hydrolysis occurred immediately after dissolving the compound in D_2_O, as shown by the immediate color fading of the solution, while ^1^H NMR spectra that were recorded instantly after dissolving the complex only show hydrolysis products. Therefore, isolation or detection of hydrolysis intermediates and time-dependent analysis is highly restricted. To understand why there is such a significant difference in water stability, we modeled and compared the hydrolysis mechanisms of both the Cp and Cp* derivatives using DTF calculations. The optimized geometries were calculated at the B3LYP/Def2-TZVP level of theory using the SMD solvation model with water as the solvent.^38^ For the Cp derivative **Cp-TSCN**, which has a κ^3^ *N*,*N* ^α^,*S* coordination mode,^23^ there are two possible hydrolysis paths; the attack of a water molecule occurs either at the sulfur site, leading to an intermediate with a κ^1^ *S* coordination of the TSCN (**Cp-S**), or at the pyridine site with a κ^1^ *N* intermediate (**Cp-N**) (Scheme 4).

**Scheme 4:**
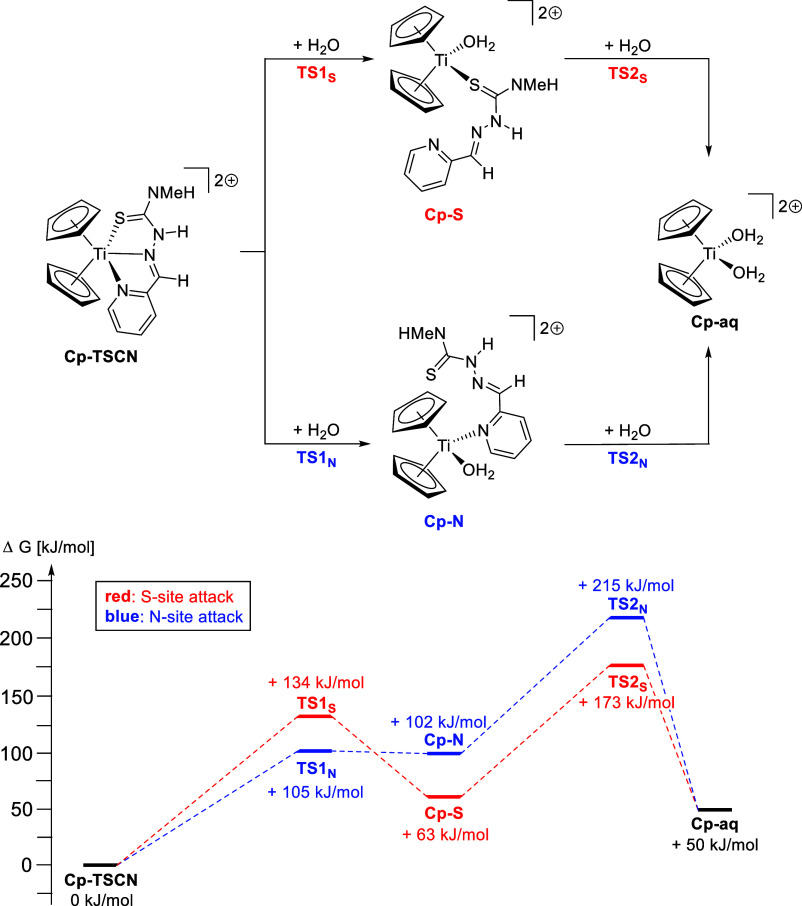
Top: Proposed Hydrolysis Reaction Paths of Titanocene-Based Complexes with the TSCN Ligand^a^

a Bottom: calculated relative free energies of intermediates and transition states along the reaction paths.

The activation energy toward **Cp-S** with +134 kJ/mol is higher than for **Cp-N** with +101 kJ/mol; however, **Cp-S** is the more stable intermediate with +63 kJ/mol in comparison to Cp-N and +102 kJ/mol relative to the starting complex **Cp-TSCN**. The transition state of **TS1**_S_ indicates an associative mechanism in which a κ^2^ *N* ^α^,*S* coordination mode is formed, leading to a more stable intermediate. In the case of **TS1**_N_, a κ^2^ *N*,*N* ^α^ coordination mode is formed during the attack of the water molecule, which is best described as an associative interchange mechanism due to the unstable intermediate.^40^ After the attack of a second water molecule, TSCN is released, while the previously observed diaqua complex **Cp-aq** ([Cp_2_Ti­(H_2_O)_2_]^2+^) is formed. The activation energies, relative to intermediates **Cp-S** and **Cp-N**, are again rather high with +110 kJ/mol and +113 kJ/mol, respectively, while the hydrolysis product **Cp-aq** with 50 kJ/mol relative to the starting complex **Cp-TSCN** is thermodynamically unfavored in the overall reaction. Again, we observe an associative mechanism in these reactions as the transition states show that both water molecules and either the sulfur atom (**TS2**_S_) or the pyridine (**TS2**_N_) are bound. The reason for the observed hydrolysis despite the thermodynamic stability of the end product is most likely the insolubility of the free TSCN in water. Therefore, upon formation of free TSCN, it is removed from the equilibrium of this reaction, which consequently shifts the equilibrium toward the product side and promotes the hydrolysis.

In contrast to the Cp derivative, the Cp* variant **Ti3a** ^**2+**^ undergoes a different hydrolysis reaction due to the divergent coordination mode (Scheme 5). In this case, the attack of a water molecule leads to the formation of an aqua complex with maintained κ^2^ *N*,*S* coordination Cp*-N with +107 kJ/mol. The transition state of this reaction **TS1**_N_ * has a slightly lower energy (+114 kJ/mol) when compared with the above calculations on the Cp derivatives and is most likely possible at room temperature. Deprotonation of the aqua-ligand by the coordinating nitrogen atom leads to a second, more stabilized κ^1^ *S* intermediate Cp*-N2 with +20 kJ/mol, where the protonated N atom bonds to the hydroxyl group. The second attack of a water molecule leads to the final product of the hydrolysis: the diaqua-complex Cp*-aq, and free TSCN with +37 kJ/mol, while the respective transition state **TS2**_N_ * (+75 kJ/mol) is even lower than the previous one. Another possible mechanism via the sulfur side was also considered (**ESI**, Table S6); however, this path contains higher activation barriers in comparison with the nitrogen site reaction and is therefore unfavored. Despite the fact that the final product is thermodynamically higher than the starting complex, the hydrolysis reaction is triggered by the release of the insoluble TSCN, which then exits the equilibrium of this reaction. While the relative activation energies of both mechanisms are quite similar, the major difference between both hydrolysis reactions is the global maximum of the rate-determining steps. The second hydrolysis mechanism has an activation energy of approximately 114 kJ/mol as the global maximum, whereas the global maximum in the first mechanism reaches 173 kJ/mol for the S-side hydrolysis and 215 kJ/mol for the N-side hydrolysis. Due to the higher global maximum, the hydrolysis proceeds more slowly in the first mechanism. Additionally, the intermediates in the first mechanism are thermodynamically less favored (63 and 102 kJ/mol vs 20 kJ/mol), which increases the rate of the back reaction in the equilibrium of the second mechanism. Ultimately, the energetically low transition states and release of the insoluble TSCN explain the fast hydrolysis of the Cp* derivative. Additionally, the hydrolysis product of the Cp*-derivative Cp*-aq is energetically lower than the Cp-variant **Cp-aq** with a difference of 13 kJ/mol, meaning that the hydrolysis of the Cp*-complex is preferred thermodynamically. An alternative path via the sulfur site is less likely due to the unfavored energy profile (**ESI**, Table S7).

**Scheme 5:**
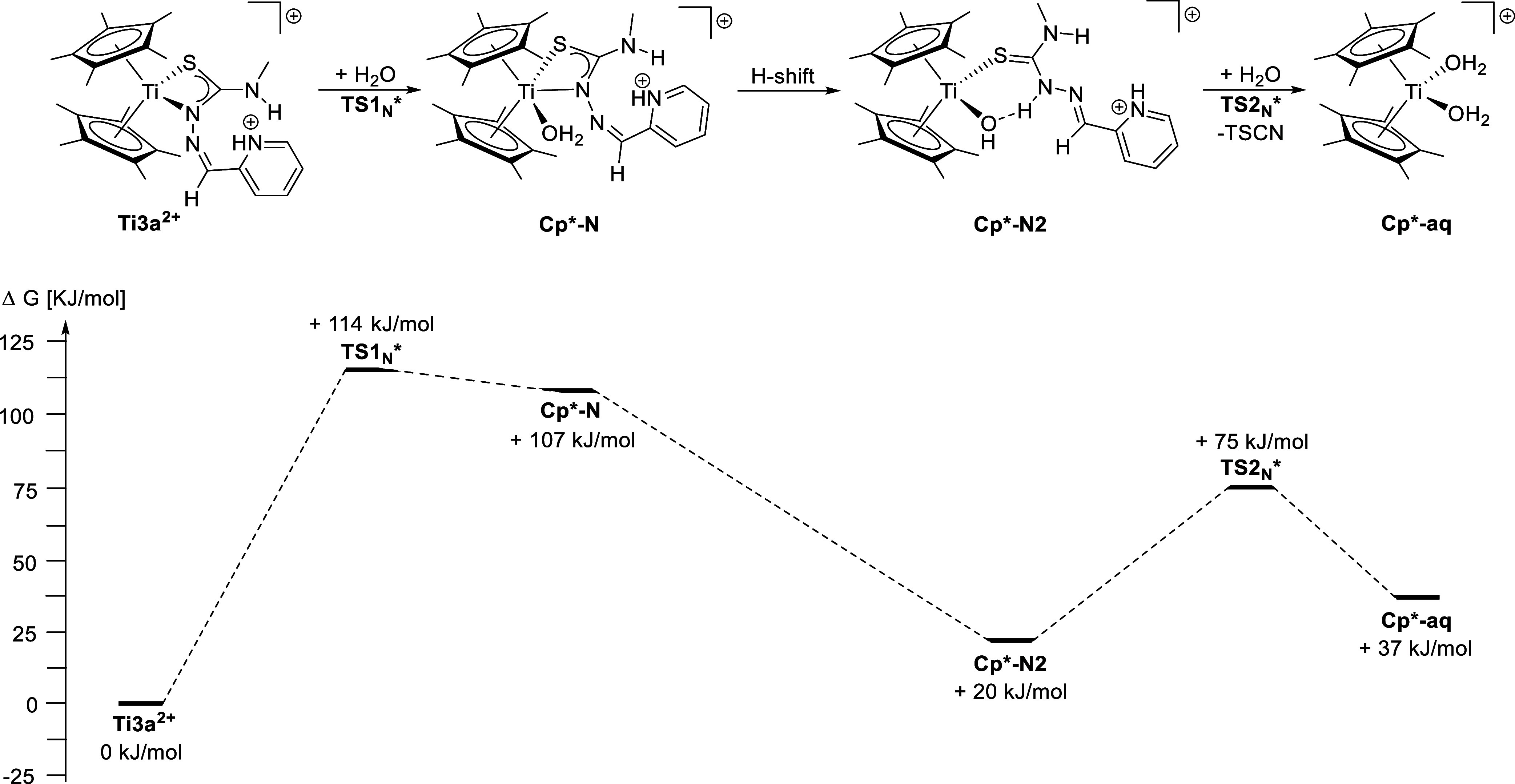
Proposed Hydrolysis Reaction of the Cp*_2_Ti-Based TSCN Complex **Ti3a** ^**2+**^

## Summary and Conclusions

In this work, we successfully synthesized the Cp* variants of titanocene-based complexes with a TSCN ligand. Crystal structures revealed surprising κ^2^ *N*,*S* coordination modes in contrast to the κ^3^ *N*,*N* ^α^,*S* coordination that has been observed for the Cp derivatives, showcasing the effect of Cp* ligands on the coordination chemistry. The free pyridine group of the TSCN ligand could then act as a proton shuttle and is protonated following the displacement of the substitution=labile triflato ligands of titanocene­(IV)­triflate with TSCN. We demonstrated with DFT calculations that an intramolecular mechanism for the proton transfer is possible in aprotic solvents, thereby explaining the unusual coordination mode that was observed in the crystal structure. We observed the instant hydrolysis of the Cp* complexes in aqueous solutions in contrast to the more stable Cp derivatives, which are stable for several hours. To explain this stark contrast, we modeled the hydrolysis reactions for the Cp and Cp* complexes using DFT calculations and found divergent mechanisms, which explains the lower water stability of the Cp* derivatives. These mechanistic studies could serve as templates for the rational design of drug carriers in the future. In summary, while the introduction of Cp* ligands resulted in higher lipophilicity and a lowered water stability, our studies gave important insights regarding coordination chemistry aspects, the mechanisms of hydrolysis reactions, and future organometallic drug carrier design.

## Experimental Section

All reactions were carried out under a dry nitrogen or argon atmosphere using standard Schlenk and glovebox techniques. Solvents were dried according to standard procedures over Na/K alloy with benzophenone as an indicator and subsequently distilled and stored under a nitrogen atmosphere. Cp*_2_Ti­(BTMSA),^41^ Cp*_2_Ti­(OTf)_2_,^42^ Fc­(OTf),^43^ and TSCN **a** ^44^ were prepared according to general (modified) methods and published procedures. Cp*_2_Ti­(OTf)_2_ was prepared with strict exclusion of water and oxygen to avoid the formation of aquacomplexes. EPR spectra were recorded on a Magnettech ESR spectrometer model MS300. NMR spectra were recorded on a Bruker AVANCE III 500 or a JEOL JNM-ECZL500R spectrometer (^1^H 500 MHz). IR spectra were recorded on a Shimadzu IR Spirit T spectrometer by using an attenuated total reflection (ATR) method. Elemental analyses were carried out on a Euro EA 3000 elemental analyzer. Melting points were determined using a Mettler Toledo MP30 instrument. High-resolution mass spectra were measured on a Thermo Scientific Exploris 240 spectrometer in aqueous solutions by using ESI. Further exact details of EPR spectra (**S2**), NMR spectra (**S3**–**S7**), crystallographic data (**S7**–**S10**), IR spectra (**S11**, **S12**), and computational details (**S13**–**S34**) are given in the Supporting Information (**ESI**).

### Syntheses and Characterization

#### Synthesis of **Ti1a**

Di­(pentamethylcyclopentadienyl)­titanium bis­(trimethylsilyl)­acetylene **Ti1** (300 mg, 0.614 mmol) and benzaldehyde *N*-methylthiosemicarbazone **a** (119 mg, 0.614 mmol) were dissolved in 10 mL of dry THF. The reaction mixture was stirred for 6 h at 60 °C to give a dark yellow green solution. The reaction mixture was stirred for additional 16 h at room temperature. The solvent was removed under reduced pressure, and the residue was washed with *n*-hexane (3 × 10 mL). All volatile components were removed under reduced pressure, and the residue was dried under vacuum to yield the product as a brown solid. Green crystals suitable for single crystal X-ray diffraction analysis precipitated from a saturated solution of **Ti1a** in CD_2_Cl_2_/*n*-hexane at −20 °C after several days. Yield: 0.291 g, 0.569 mmol, 93%. IR (ATR): ṽ = 3390, 3291 (N–H), 2962, 2904, 2854, 1580 (Py), 1520, 1467, 1432, 1375, 1366, 1322, 1273, 1247, 1147, 1099, 1072, 1021, 988, 943, 843, 799, 777, 743, 671, 618, 583, 539, 503 cm^–1^. Mp. 171–179 °C (dec.). EPR: *g* = 1.976. HR/MS: calcd: *m*/*z* = 511.2375 [M^+^], measured (ESI): *m*/*z* = 511.2374. EA: calcd for C_28_H_39_N_4_STi: C 65.74, H 7.68, N 10.95; found, C 65.46, H 7.41, N 10.24.

#### Synthesis of **Ti2a**

Complex **Ti1a** (200 mg, 0.391 mmol) and ferrocenium triflate (131 mg, 0.391 mmol) were dissolved in 10 mL of dry THF. The reaction mixture was stirred for 2 h at room temperature to give a red solution. The reaction mixture was then diluted with 10 mL of *n*-hexane. A green solid precipitated, and the supernatant was decanted. The residue was washed with *n*-hexane (2 × 10 mL). All volatile components were removed under reduced pressure, and the residue was dried under vacuum to yield the product as a green solid. Green crystals suitable for single-crystal X-ray diffraction analysis precipitated from a saturated solution of **Ti2a** in CD_2_Cl_2_/*n*-hexane at −20 °C after several days. Yield: 0.124 g, 0.188 mmol, 48%. ^1^H NMR (500 MHz, CD_2_Cl_2_, 298 K): δ = 2.06 (s, 30H, Cp*-Me), 3.22 (d, 3H, N–Me), 7.23–7.28 (m, 1H, Ar–H), 7.71–7.76 (m, 1H, Ar–H), 7.76–7.84 (m, 1H, Ar–H), 8.14–8.18 (m, 1H, Ar–H) 8.49 (d, 1H, aldimine-H), 8.85–8.89 (m, 1H, Ar–H) ppm. The N–H signal was not detected. ^13^C­{^1^H} NMR (125 MHz, CD_2_Cl_2_, 298 K): δ = 13.0 (10 x Cp*-CH_3_), 26.0 (N–Me), 120.2 (Ar–C_q_), 122.7 (Ar–CH), 124.8 (10 x Cp*-C_q_), 126.3 (Ar–CH), 137.1 (Ar–CH), 149.9 (aldimine-CH), 152.5 (Ar–CH) ppm. ^19^F­{^1^H} NMR (470 MHz, CD_2_Cl_2_, 298 K): δ = −78.8 ppm. IR (ATR): ṽ = 3267 (N–H), 2906, 1584 (Py), 1491, 1466, 1427, 1380, 1330, 1306, 1278, 1254, 1223, 1145, 1102, 1083, 1028, 991, 945, 804, 780, 755, 746, 676, 636, 619, 571, 515 cm^–1^. Mp. 188–192 °C (dec.). HR/MS: calculated: *m*/*z* (cation) = 511.2375 [M^+^], measured (ESI): *m*/*z* = 511.2379; calcd: *m*/*z* (anion) = 148.9520 [M^–^], measured (ESI): *m*/*z* = 148.9527. EA: calcd for C_29_H_39_F_3_N_4_S_2_O_3_Ti: C 52.72, H 5.95, N 8.48; found, C 51.92, H 6.34, N 8.20.

#### Synthesis of **Ti3a**

Di­(pentamethylcyclopentadienyl)­titanium­(IV)­triflate **Ti3** (200 mg, 0.324 mmol) and 2-pyridinecarboxaldehyde *N*-methylthiosemicarbazone **a** (63 mg, 0.324 mmol) were dissolved in 10 mL of dry THF. The reaction mixture was stirred for 6 h at 60 °C to give a dark yellow solution. The reaction mixture was stirred for additional 16 h at room temperature. The reaction mixture was then diluted with 10 mL of *n*-hexane. A green solid precipitated, and the supernatant was decanted. The residue was washed with *n*-hexane (2 × 10 mL). All volatile components were removed under reduced pressure, and the residue was dried under vacuum to yield the product as a green solid. Yellow crystals suitable for single-crystal X-ray diffraction analysis precipitated from a THF solution of **Ti3a** at −20 °C after several days. Yield: 132 mg, 0.163 mmol, 50%. ^1^H NMR (500 MHz, THF-*d*_8_, 298 K): δ = 2.14 (s, 30H, Cp*-Me), 3.17 (d, *J* = 4.7 Hz, 3H, N–Me), 8.07 (s, 1H, aldimine-H), 8.13–8.18 (m, 1H, Ar–H), 8.48–8.56 (m, 1H, Ar–H), 8.80–8.85 (m, 1H, Ar–H), 8.92–8.96 (m, 1H, Ar–H) ppm. The N–H signals were not detected. No ^13^C­{^1^H} NMR spectrum is discussed due to the second set of signals of the sample and the poor solubility of the compound. ^19^F­{^1^H} NMR (470 MHz, CD_2_Cl_2_, 298 K): δ = −79.2 ppm. IR (ATR): ṽ = 3253 (N–H), 3115, 3066, 2907, 2863, 1619 (Py–H), 1582, 1576, 1557, 1554, 1539 (Py-H), 1520, 1462, 1432, 1383, 1338, 1231, 1212, 1151, 1119, 1088, 1044, 1021, 891, 886, 806, 770, 759, 668, 635, 571, 549, 538, 531, 514, 500, 495, 490, 484 cm^–1^. Mp. 105–111 °C (dec.). HR/MS: calcd: *m*/*z* (cation) = 511.2375 [M-H]^+^, measured (ESI): *m*/*z* = 511.2378; calcd: *m*/*z* (anion) = 148.9520 [M^–^], measured (ESI): *m*/*z* = 148.9528. Elemental analysis was omitted due to the secondary species overserved in the NMR spectra.

## Supplementary Material









## Notes

EPR, NMR, IR spectra, crystallographic data, and computational details (PDF)
Crystallographic information of **Ti1a** (CIF)
Crystallographic information of **Ti2a** (CIF)
Crystallographic information of **Ti3a** (CIF)

## References

[1] Tibbitt M. W., Dahlman J. E., Langer R. (2016). Emerging Frontiers in Drug Delivery. J. Am. Chem. Soc..

[2] Tiwari G., Tiwari R., Sriwastawa B., Bhati L., Pandey S., Pandey P., Bannerjee S. K. (2012). Drug delivery systems: An updated
review. Int. J. Pharm. Investig..

[3] Vargason A. M., Anselmo A. C., Mitragotri S. (2021). The evolution
of commercial drug
delivery technologies. Nat. Biomed. Eng..

[4] Li C., Wang J., Wang Y., Gao H., Wei G., Huang Y., Yu H., Gan Y., Wang Y., Mei L., Chen H., Hu H., Zhang Z., Jin Y. (2019). Recent progress
in drug delivery. Acta Pharm. Sin. B.

[5] Kingsley J. D., Dou H., Morehead J., Rabinow B., Gendelman H. E., Destache C. J. (2006). Nanotechnology:
a focus on nanoparticles as a drug
delivery system. J. Neuroimmune Pharm..

[6] Chandrakala V., Aruna V., Angajala G. (2022). Review on
metal nanoparticles as
nanocarriers: current challenges and perspectives in drug delivery
systems. Emergent Mater..

[7] Jain A., Jain A., Gulbake A., Shilpi S., Hurkat P., Jain S. K. (2013). Peptide and protein
delivery using new drug delivery
systems. Crit. Rev. Ther. Drug Carrier Syst..

[8] Elzoghby A. O., Samy W. M., Elgindy N. A. (2012). Protein-based
nanocarriers as promising
drug and gene delivery systems. J. Controlled
Release.

[9] Mjos K. D., Orvig C. (2014). Metallodrugs in medicinal
inorganic chemistry. Chem. Rev..

[10] Wang X., Wang X., Jin S., Muhammad N., Guo Z. (2019). Stimuli-Responsive
Therapeutic Metallodrugs. Chem. Rev..

[11] Anthony E. J., Bolitho E. M., Bridgewater H. E., Carter O. W. L., Donnelly J. M., Imberti C., Lant E. C., Lermyte F., Needham R. J., Palau M., Sadler P. J., Shi H., Wang F.-X., Zhang W.-Y., Zhang Z. (2020). Metallodrugs are unique:
opportunities
and challenges of discovery and development. Chem. Sci..

[12] Rosenberg B., Vancamp L., Krigas T. (1965). Inhibition of Cell Division in Escherichia
Coli by Electrolysis Products from a Platinum Electrode. Nature.

[13] Ghosh S. (2019). Cisplatin:
The first metal based anticancer drug. Bioorg.
Chem..

[14] Cini M., Bradshaw T. D., Woodward S. (2017). Using titanium
complexes to defeat
cancer: the view from the shoulders of titans. Chem. Soc. Rev..

[15] Köpf H., Köpf-Maier P. (1979). Titanocene dichloride--the first metallocene with cancerostatic
activity. Angew. Chem., Int. Ed. Engl..

[16] Lümmen G., Sperling H., Luboldt H., Otto T., Rübben H. (1998). Phase II trial
of titanocene dichloride in advanced renal-cell carcinoma. Cancer Chemother. Pharmacol..

[17] Schilling T., Keppler K. B., Heim M. E., Niebch G., Dietzfelbinger H., Rastetter J., Hanauske A. R. (1995). Clinical phase I and pharmacokinetic
trial of the new titanium complex budotitane. Invest. New Drugs.

[18] Tshuva E. Y., Ashenhurst J. A. (2009). Cytotoxic Titanium­(IV) Complexes: Renaissance. Eur. J. Inorg. Chem..

[19] Tzubery A., Tshuva E. Y. (2011). Trans titanium­(IV) complexes of salen ligands exhibit
high antitumor activity. Inorg. Chem..

[20] Tzubery A., Tshuva E. Y. (2012). Cytotoxicity and
hydrolysis of trans-Ti­(IV) complexes
of salen ligands: structure-activity relationship studies. Inorg. Chem..

[21] Tzubery A., Tshuva E. Y. (2017). Cytotoxic Titanium­(IV) Complexes of Salalen-Based Ligands. Eur. J. Inorg. Chem..

[22] Loza-Rosas S. A., Vázquez-Salgado A. M., Rivero K. I., Negrón L. J., Delgado Y., Benjamín-Rivera J. A., Vázquez-Maldonado A. L., Parks T. B., Munet-Colón C., Tinoco A. D. (2017). Expanding the Therapeutic
Potential of the Iron Chelator Deferasirox in the Development of Aqueous
Stable Ti­(IV) Anticancer Complexes. Inorg. Chem..

[23] Schwitalla K., Müller M.-C., Fabra D., Schmidtmann M., Meyer U., Matesanz A. I., Quiroga A. G., Rauch B. H., Beckhaus R. (2025). Water-Soluble, Titanocene-Based
Prodrugs with Thiosemicarbazones
as Cytotoxic Agents. ACS Omega.

[24] Kasuga N. C., Sekino K., Ishikawa M., Honda A., Yokoyama M., Nakano S., Shimada N., Koumo C., Nomiya K. (2003). Synthesis,
structural characterization and antimicrobial activities of 12 zinc­(II)
complexes with four thiosemicarbazone and two semicarbazone ligands. J. Inorg. Biochem..

[25] Netalkar P. P., Netalkar S. P., Revankar V. K. (2015). Transition metal
complexes of thiosemicarbazone:
Synthesis, structures and invitro antimicrobial studies. Polyhedron.

[26] Nartop D., Hasanoğlu Özkan E., Öğütcü H., Kurnaz Yetim N., Özdemir I. ˙. (2024). Novel α-N-heterocyclic thiosemicarbazone
complexes: synthesis, characterization, and antimicrobial of properties
investigation. RSC Adv..

[27] Pelosi G., Bisceglie F., Bignami F., Ronzi P., Schiavone P., Re M. C., Casoli C., Pilotti E. (2010). Antiretroviral activity
of thiosemicarbazone metal complexes. J. Med.
Chem..

[28] Kesel A. J. (2011). Broad-spectrum
antiviral activity including human immunodeficiency and hepatitis
C viruses mediated by a novel retinoid thiosemicarbazone derivative. Eur. J. Med. Chem..

[29] Padmanabhan P., Khaleefathullah S., Kaveri K., Palani G., Ramanathan G., Thennarasu S., Tirichurapalli Sivagnanam U. (2017). Antiviral
activity
of Thiosemicarbazones derived from α-amino acids against Dengue
virus. J. Med. Virol..

[30] Padhye S., Afrasiabi Z., Sinn E., Fok J., Mehta K., Rath N. (2005). Antitumor metallothiosemicarbazonates: structure and antitumor activity
of palladium complex of phenanthrenequinone thiosemicarbazone. Inorg. Chem..

[31] Huang H., Chen Q., Ku X., Meng L., Lin L., Wang X., Zhu C., Wang Y., Chen Z., Li M., Jiang H., Chen K., Ding J., Liu H. (2010). A series of
alpha-heterocyclic carboxaldehyde thiosemicarbazones inhibit topoisomerase
IIalpha catalytic activity. J. Med. Chem..

[32] Palanimuthu D., Shinde S. V., Somasundaram K., Samuelson A. G. (2013). In vitro
and in vivo anticancer activity of copper bis­(thiosemicarbazone) complexes. J. Med. Chem..

[33] Hidalgo T., Fabra D., Allende R., Matesanz A. I., Horcajada P., Biver T., Quiroga A. G. (2023). Two novel Pd thiosemicarbazone complexes
as efficient and selective antitumoral drugs. Inorg. Chem. Front..

[34] Melones-Herrero J., Aguilar-Rico F., Matesanz A. I., Calés C., Sánchez-Pérez I., Quiroga A. G. (2025). Antiproliferative
activity in breast cancer cells of PtL2: A steroid-thiosemicarbazone
platinum­(II) complex. J. Inorg. Biochem..

[35] Casali E., Gandini A., Merlo G., Carli L., Weigand J. J., Porta A., Zanoni G. (2025). Titanocenes
functionalization with
high chemical diversity via titanium protecting groups. Commun. Chem..

[36] Rosenthal U. (2020). Equilibria
and mesomerism/valence tautomerism of group 4 metallocene complexes. Chem. Soc. Rev..

[37] Schwitalla K., Yusufzadeh Z., Schmidtmann M., Beckhaus R. (2024). From Coordination to
Noncoordination: Syntheses and Substitution Lability Studies of Titanium
Triflato Complexes. Inorg. Chem..

[38] Marenich A. V., Cramer C. J., Truhlar D. G. (2009). Universal
solvation model based on
solute electron density and on a continuum model of the solvent defined
by the bulk dielectric constant and atomic surface tensions. J. Phys. Chem. B.

[39] Tolman C. A. (1972). The 16
and 18 electron rule in organometallic chemistry and homogeneous catalysis. Chem. Soc. Rev..

[40] Casali E., Toma L., Porta A., Weigand J. J., Zanoni G. (2025). Mechanism
of Ligand Exchange Processes for Titanocene Complexes: A Computational
Study. Organometallics.

[41] Varga V., Mach K., Polášek M., Sedmera P., Hiller J., Thewalt U., Troyanov S. I. (1996). Titanocene-bis­(trimethylsilyl)­acetylene
complexes: effects of methyl substituents at the cyclopentadienyl
ligands on the structure of thermolytic products. J. Organomet. Chem..

[42] Thewalt U., Honold B. (1988). Darstellung und Kristallstrukturen
von Salzen mit den
Kationen [Cp*_2_ Ti­(H_2_O)_2_]^2+^ und [Cp*_2_Ti­(OH)­(H_2_O)] + (Cp* = η^5^-(CH_3_)_5_C_5_). J. Organomet. Chem..

[43] Adhikari D., Mossin S., Basuli F., Huffman J. C., Szilagyi R. K., Meyer K., Mindiola D. J. (2008). Structural, spectroscopic,
and theoretical
elucidation of a redox-active pincer-type ancillary applied in catalysis. J. Am. Chem. Soc..

[44] Jiang M., Chu Y., Yang T., Li W., Zhang Z., Sun H., Liang H., Yang F. (2021). Developing a Novel Indium­(III) Agent
Based on Liposomes to Overcome Cisplatin-Induced Resistance in Breast
Cancer by Multitargeting the Tumor Microenvironment Components. J. Med. Chem..

