# Supplementary material for: Effects of Cp*-Ligands in Titanocene-Based Thiosemicarbazone Complexes
Source: ACS Omega. 2026 May 14;11(20):30123–30. doi: 10.1021/acsomega.6c02122 (PMC13216990; doi:10.1021/acsomega.6c02122)
Supplement: Supplementary file 1 [file ao6c02122_si_001.pdf]

Supporting Information

Effects of Cp* Ligands in Titanocene-based Thiosemicarbazone Complexes

Kevin Schwitalla*[a] and Marc Schmidtmann[a],

[a] Chemistry Department, Carl von Ossietzky University of Oldenburg, 26111 Oldenburg, Germany

TABLE OF CONTENTS

EPR spectra...... 2

NMR Spectra of complexes 3

NMR Spectra of water-solubility and stability studies 6

Crystallographic data...... 7

IR spectra...... 11

Computational Details...... 13

References...... 35

EPR Spectra

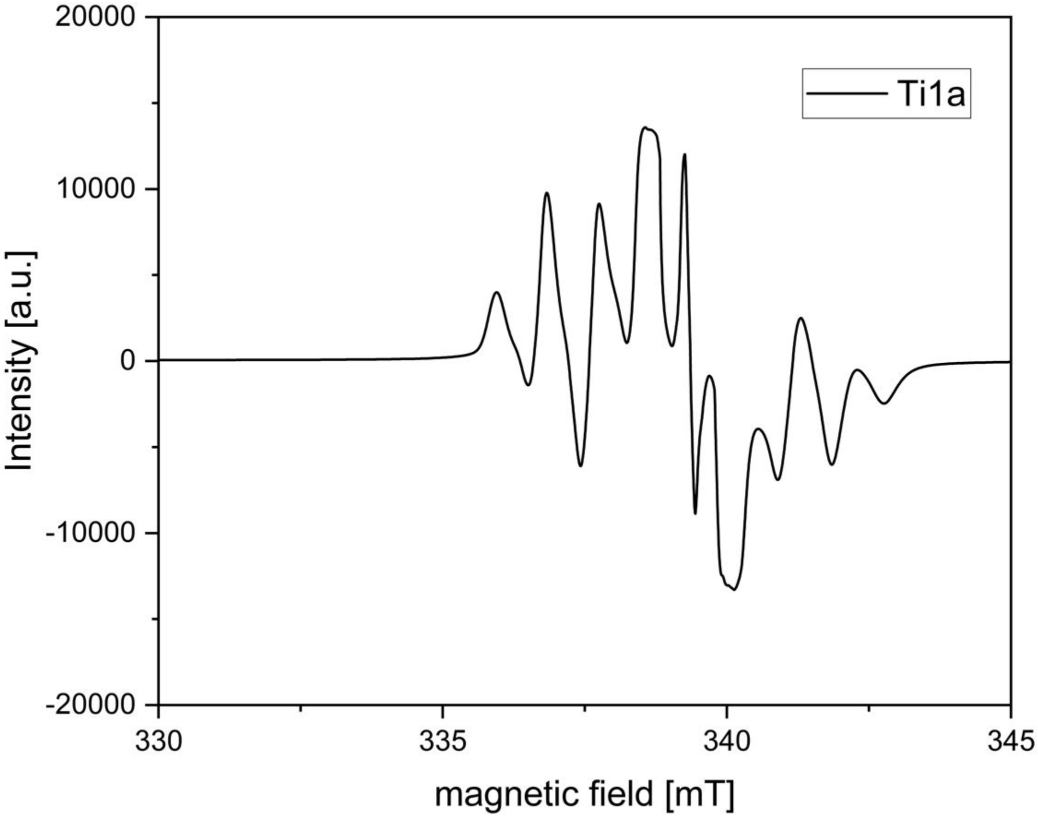

Figure S1: EPR spectrum of complex **Ti1a** in benzene at room temperature (g = 1.976).

NMR Spectra of complexes

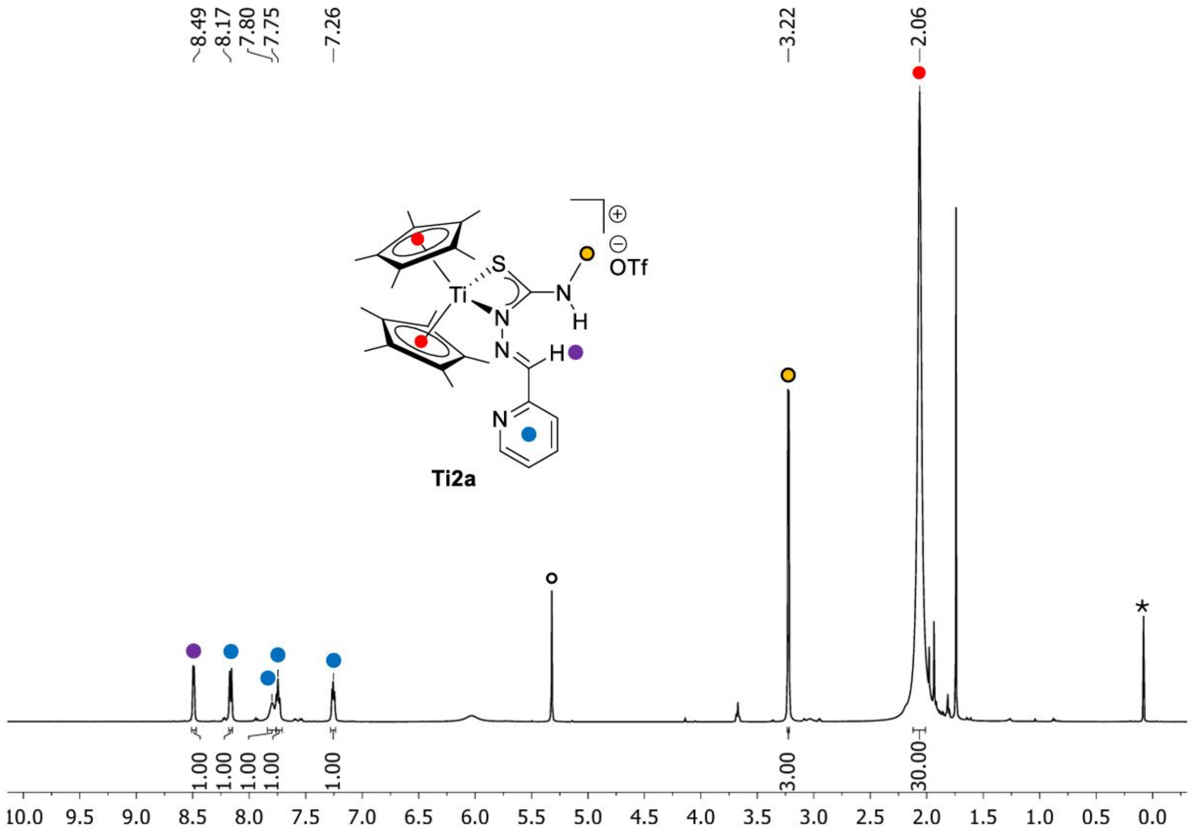

Figure S2: ^{1}H NMR spectrum (500 MHz, CD_2Cl_2, 298 K) of **Ti2a**. Product signals given in colors (° = $CHDCl_2$, * = grease).

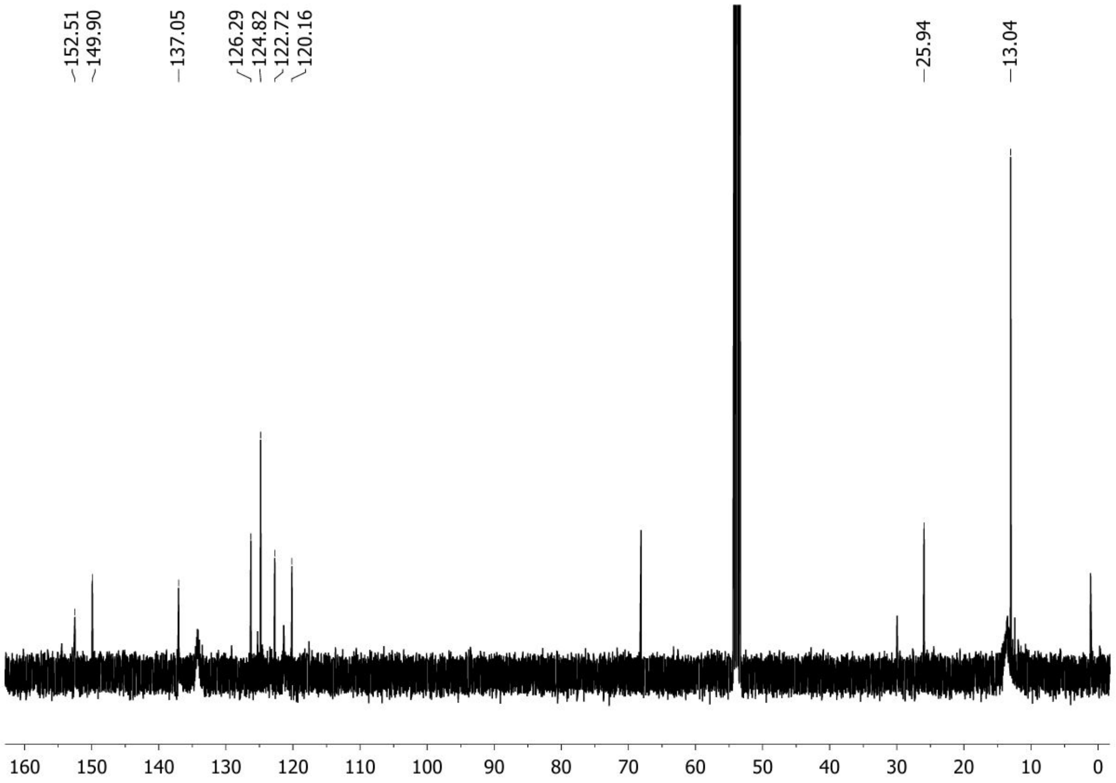

Figure S3: $^{13}C\{^{1}H\}$ NMR spectrum (125 MHz, CD_2Cl_2, 298 K) of **Ti2a**.

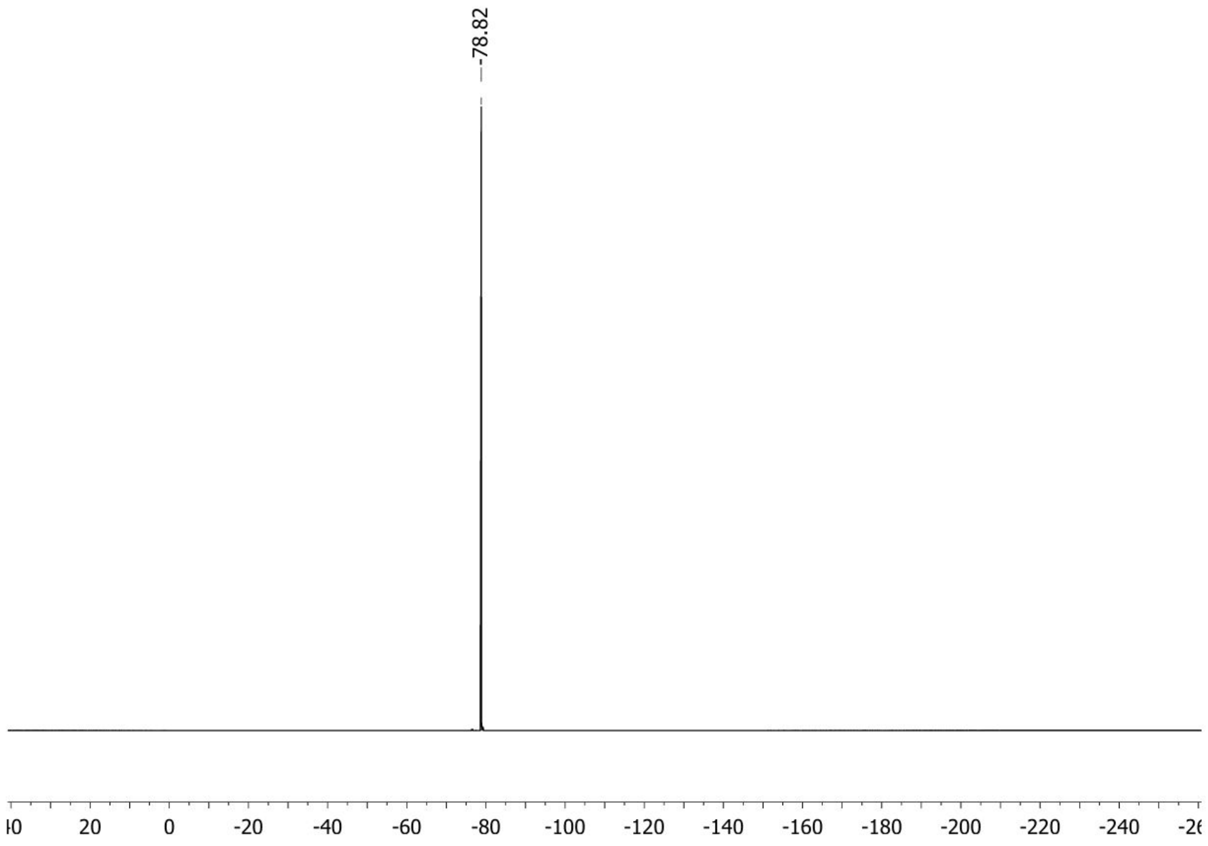

Figure S4: $^{19}F\{^{1}H\}$ NMR spectrum (470 MHz, CD_2Cl_2, 298 K) of **Ti2a**.

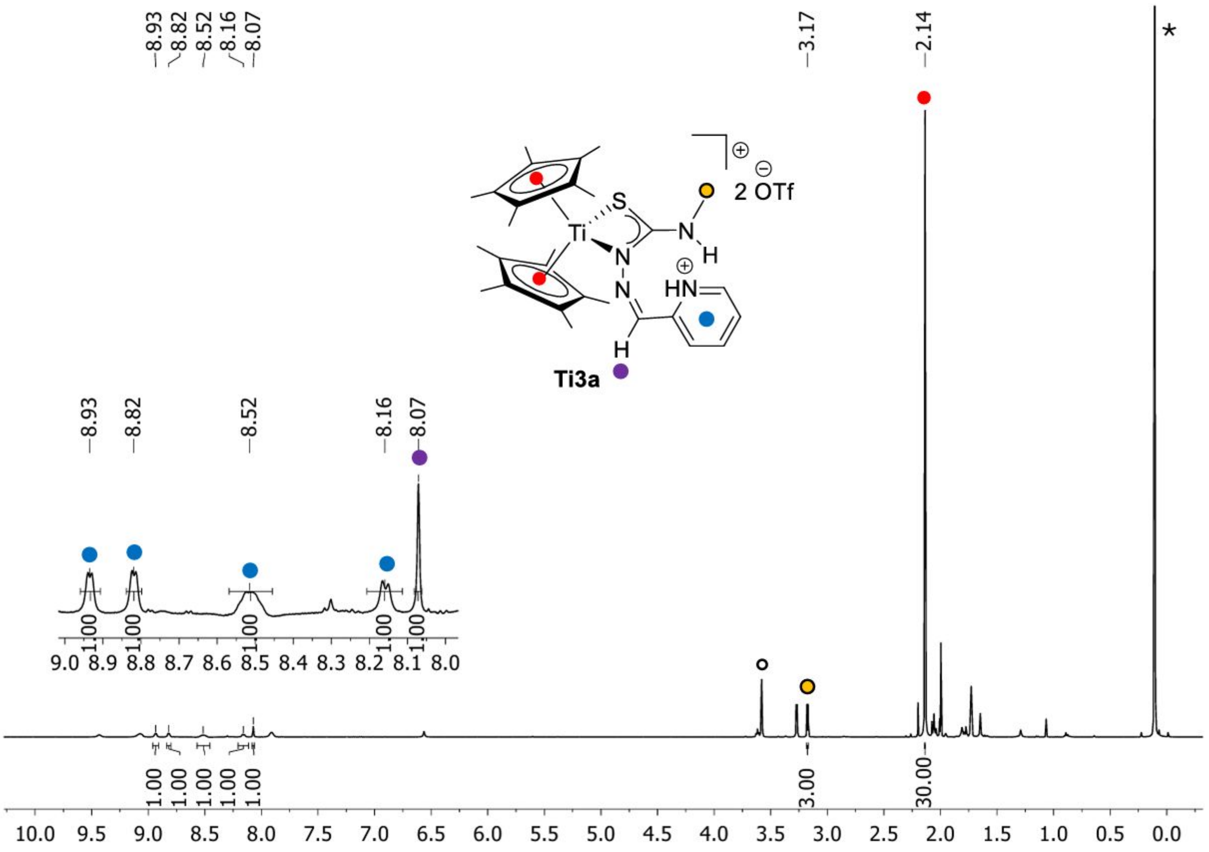

Figure S5: ^{1}H NMR spectrum (500 MHz, THF-*d*8, 298 K) of **Ti3a**. Product signals given in colors (° = C_4HD_7O, * = grease).

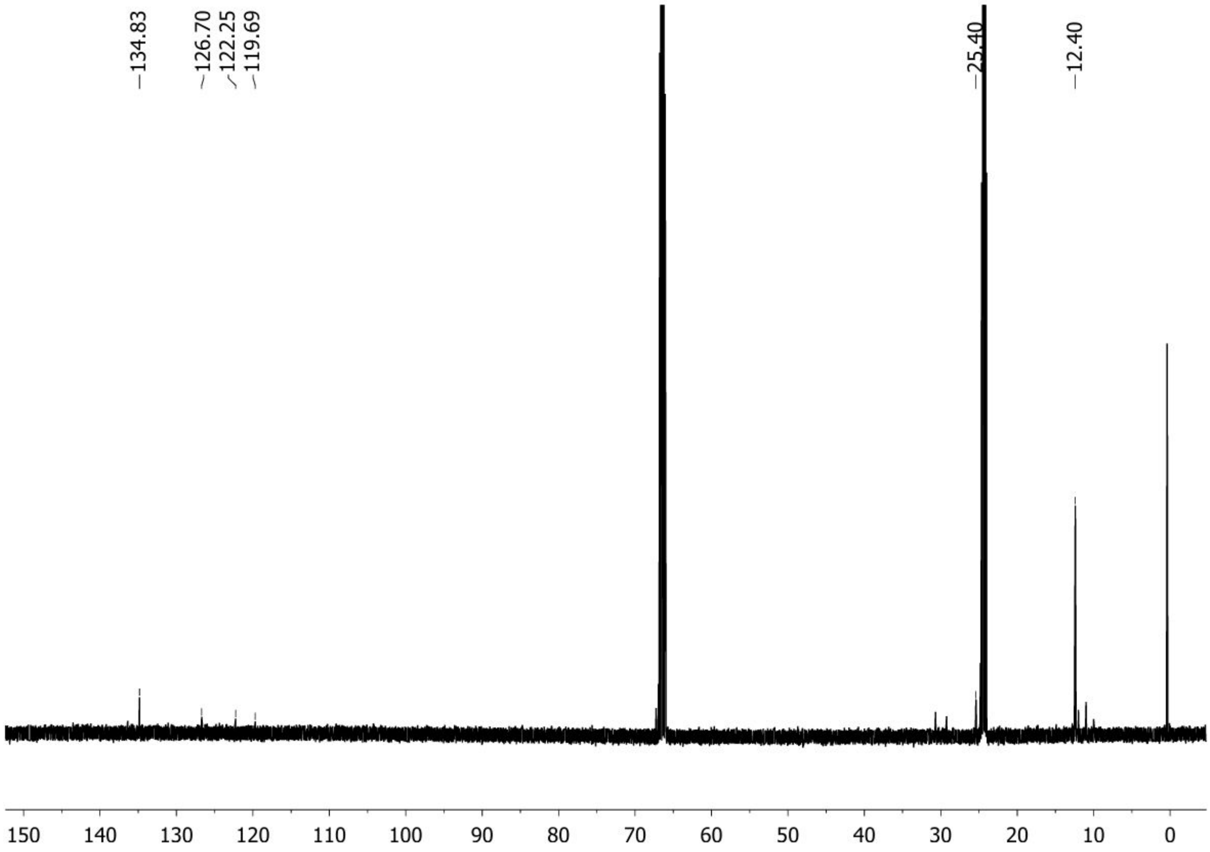

Figure S6: $^{13}C\{^{1}H\}$ NMR spectrum (125 MHz, CD_2Cl_2, 298 K) of **Ti3a**.

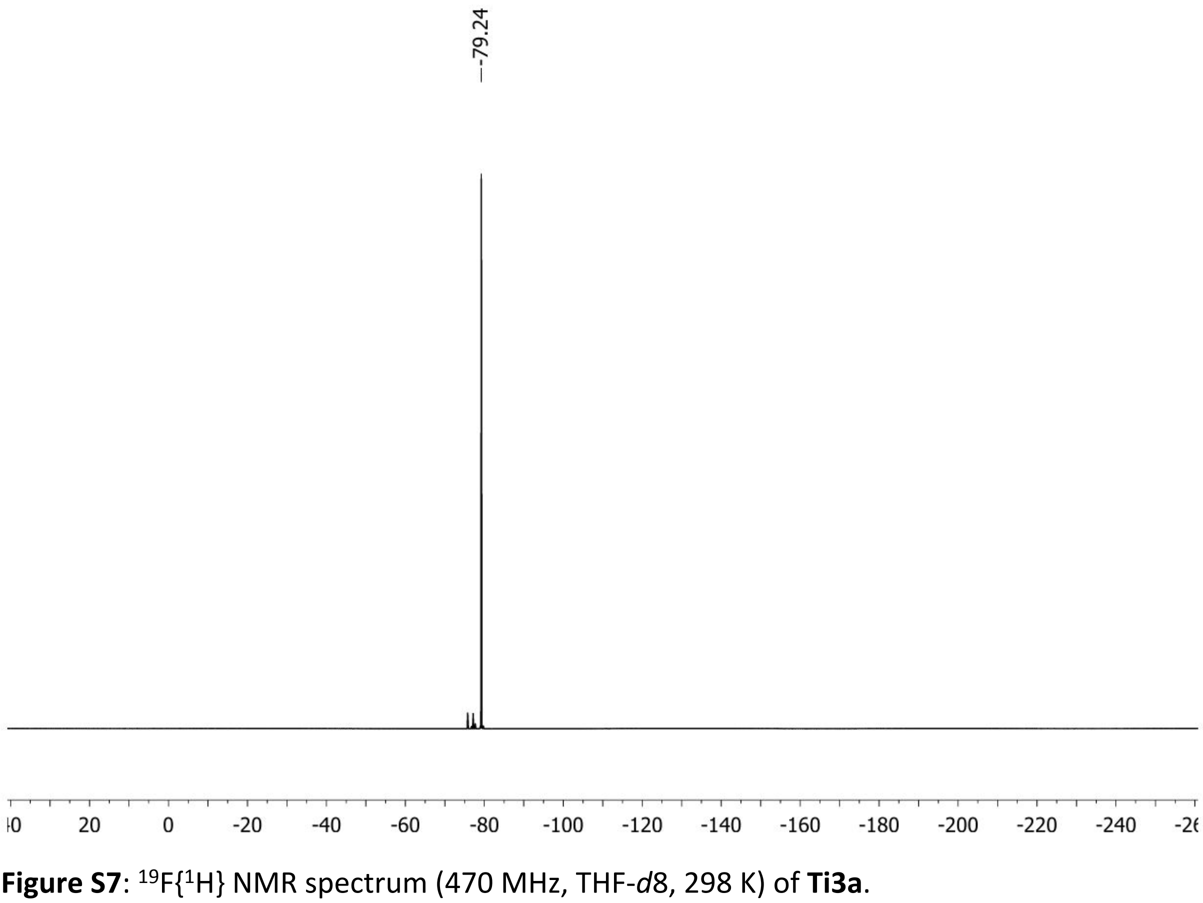

Figure S7: $^{19}F\{^{1}H\}$ NMR spectrum (470 MHz, THF-*d*8, 298 K) of **Ti3a**.

NMR Spectra of water-stability studies

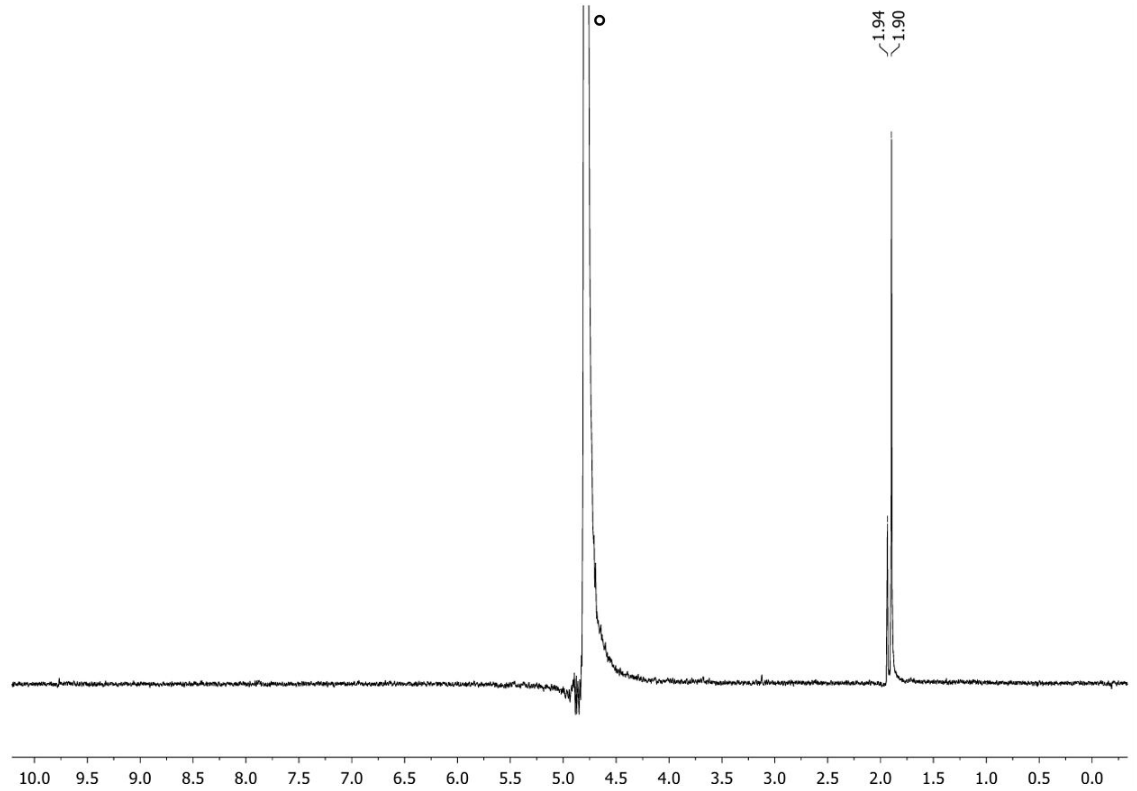

Figure S8: ^{1}H NMR spectra (300 MHz, D_2O, 298 K) of **Ti2a** over time. 1.90, 1.94 ppm: Cp* signals of hydrolyzed products (° = HDO).

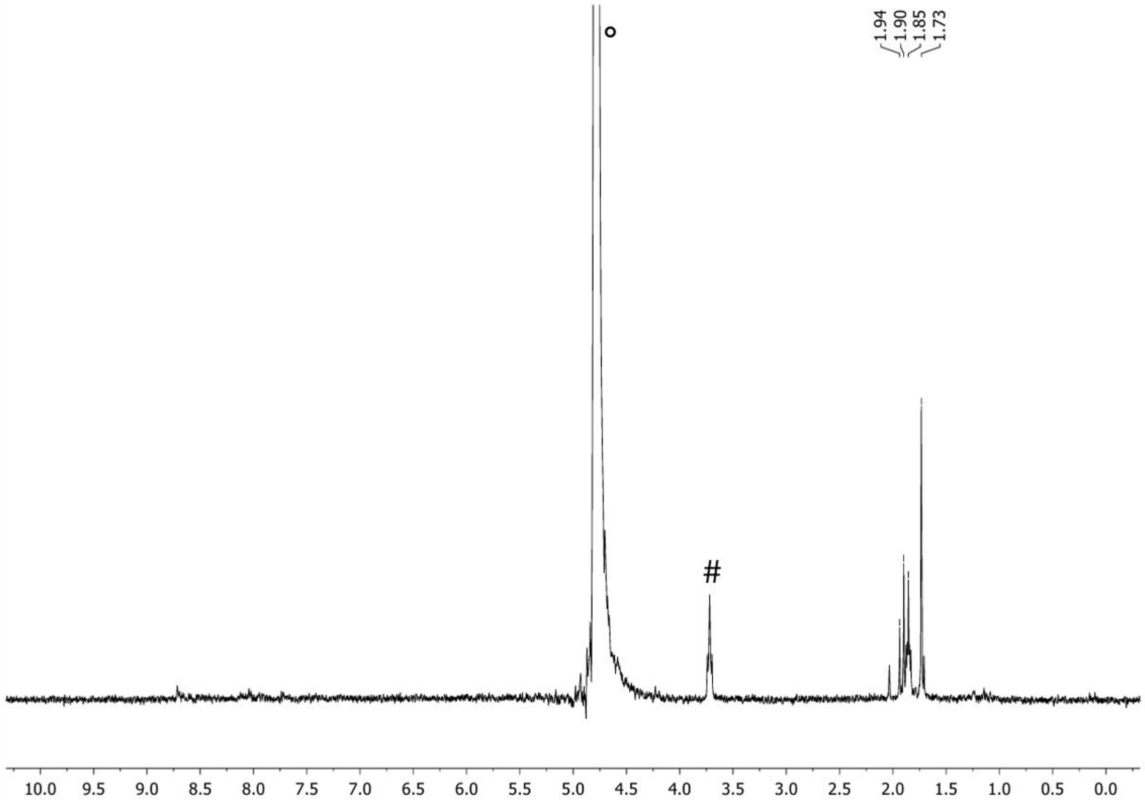

Figure S9: ^{1}H NMR spectra (300 MHz, D_2O, 298 K) of **Ti3a** over time. 1.90, 1.94 ppm: Cp* signals of hydrolyzed products (# = THF, ° = HDO).

Crystallographic data

Single crystal X-ray data were measured on a Bruker AXS D8 Venture diffractometer (multilayer optics, Mo-Kα and Cu-Kα radiation with λ = 0.71073 Å and 1.54178 Å respectively, Kappa 4-circle goniometer, Photon III C14 CPAD detector). All crystals were measured at a temperature of 100 K. Absorption corrections using equivalent reflections were performed with the program SADABS.[1]

All structures were solved with the program SHELXS[2] and refined with SHELXL[3] using the OLEX2[4] GUI.

All non-H atoms were refined with anisotropic atomic displacement parameters (ADPs); in case of the disordered Cp* ligand in **Ti1a**, the minor site (~21%) was refined with isotropic ADPs instead. H atoms bonded to C were located in the difference Fourier maps and placed on idealized geometric positions with idealized ADPs using the riding model. H atoms bonded to N were refined freely.

Ti1a contains two independent molecules in the asymmetric unit. One of them shows minor disorder of the TSCN ligand (<5%) of unknown nature. Attempts to refine the same ligand with a different coordination mode or orientation did not succeed, and the disorder has been omitted eventually from the refinement model. The disorder, however, gives rise to a couple of Hirshfeld test differences.

The crystallographic data can be obtained free of charge from https://www.ccdc.cam.ac.uk/structures/ quoting the CCDC numbers 2502783-2502785.

Table S1: Crystallographic data of **Ti1a**, **Ti2a**, **Ti3a**.

	**Ti1a**	**Ti2a**	**Ti3a**
CCDC	2502785	2502784	2502783
Lab-ID	KESC355	KESC358	KESC353
empirical formula	$C_{28}H_{39}N_4STi$	$C_{29}H_{39}F_3N_4O_3S_2Ti$	$C_{30}H_{40}F_6N_4O_6S_3Ti$
Fw	511.59	660.66	810.74
Colour	green	green	green
Habit	plate	plate	plate
cryst. dimens. mm	0.30 x 0.08 x 0.02	0.12 x 0.06 x 0.02	0.06 x 0.04 x 0.01
cryst. system	monoclinic	triclinic	triclinic
space group	C2/c	P-1	P-1
a, Å	26.8167(13)	8.6531(5)	9.2508(4)
b, Å	9.4352(4)	13.6041(9)	12.2129(5)
c, Å	42.504(2)	13.6094(9)	16.6145(7)
α, deg	90	97.085(3)	91.082(3)
β, deg	94.602(2)	103.578(3)	90.117(3)
γ, deg	90	97.450(3)	105.904(2)
V, Å^3	10719.8(9)	1524.37(17)	1804.86(13)
Z	16	2	2
$D_{calc.}$, g cm^{-3}	1.268	1.439	1.492
μ, mm^{-1}	0.420	0.474	4.326
T, K	100(2)	100(2)	100(2)
λ, Å	0.71073	0.71073	1.54178
θ range, deg	1.524 – 27.101	1.529 – 28.700	2.660 – 66.593
reflections collected	323562	102930	29753
Indep. Reflecions R(int)	11803 0.0841	7875 0.0764	6362 0.0839
Observed reflections (I > 2(I))	10490	6937	4961
Absorption correction	semi-empirical	semi-empirical	semi-empirical
max, min transm.	0.9964, 0.9548	1.0000, 0.8331	1.0000, 0.8915
final R indices [$I>2\sigma(I)$]	R1 = 0.0442, wR2 = 0.0968	R1 = 0.0623, wR2 = 0.1569	R1 = 0.0541, wR2 = 0.1344
R indices (all data)	R1 = 0.0517, wR2 = 0.1004	R1 = 0.0709, wR2 = 0.1622	R1 = 0.0719, wR2 = 0.1464
GOF on F^2	1.083	1.161	1.030
largest diff peak / hole (e.Å^{-3})	1.164 / -0.454	1.173 / -1.013	0.562 / -0.399

Molecular / crystal structures

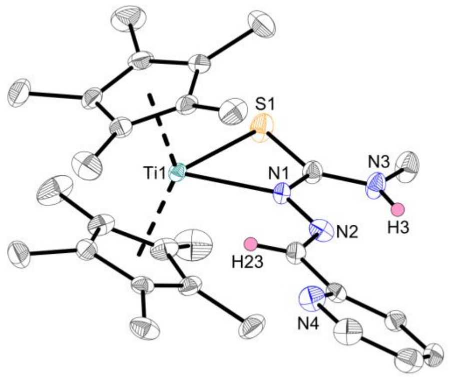

Figure S10: Molecular structure of complex **Ti1a**. Displacement ellipsoids are drawn at the 50% probability level. Redundant H atoms and solvent molecules have been omitted for clarity. Selected bond lengths (Å) and angles (deg): Ti1–N1 2.1986(16), Ti1–S1 2.5890(6), N1–N2 1.354(2), N1–Ti1–S1 64.44(5), Ti1–N1–N2 142.52(13), Ct1–Ti1–Ct2 139.7 (Ct1 = centroid of Cp*; Ct2 = centroid of Cp*).

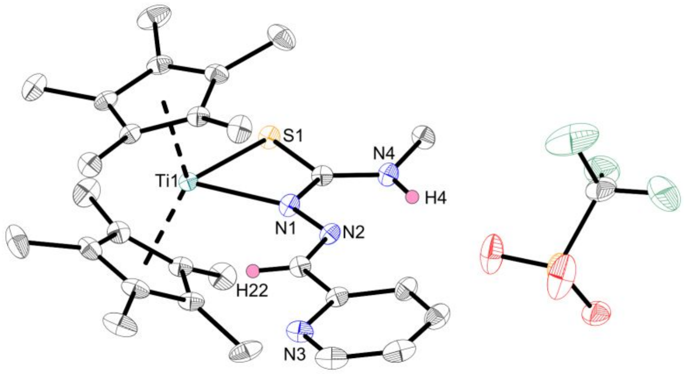

Figure S11: Molecular structure of complex **Ti2a**. Displacement ellipsoids are drawn at the 50% probability level. Redundant H atoms and solvent molecules have been omitted for clarity. Selected bond lengths (Å) and angles (deg): Ti1–N1 2.121(2), Ti1–S1 2.5265(7), N1–N2 1.382(3), N1–Ti1–S1 66.20(6), Ti1–N1–N2 142.26(16), Ct1–Ti1–Ct2 138.7 (Ct1 = centroid of Cp*; Ct2 = centroid of Cp*).

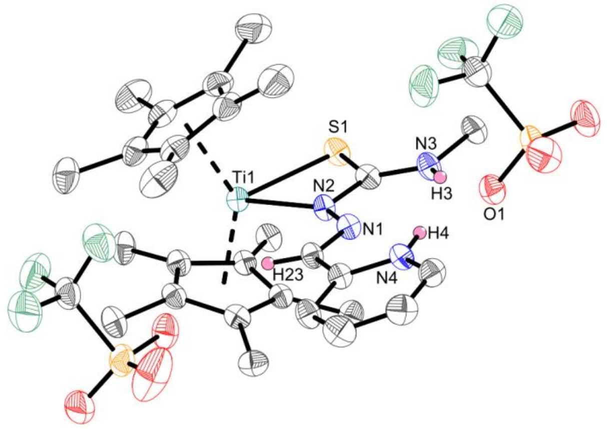

Figure S12: Molecular structure of complex **Ti3a**. Displacement ellipsoids are drawn at the 50% probability level. Redundant H atoms and solvent molecules have been omitted for clarity. Selected bond lengths (Å) and angles (deg): Ti1–N2 2.172(3), Ti1–S1 2.5145(10), N1–N2 1.355(4), N2–Ti1–S1 65.99(7), Ti1–N2–N1 145.5(2), Ct1–Ti1–Ct2 138.7 (Ct1 = centroid of Cp*; Ct2 = centroid of Cp*).

IR spectra

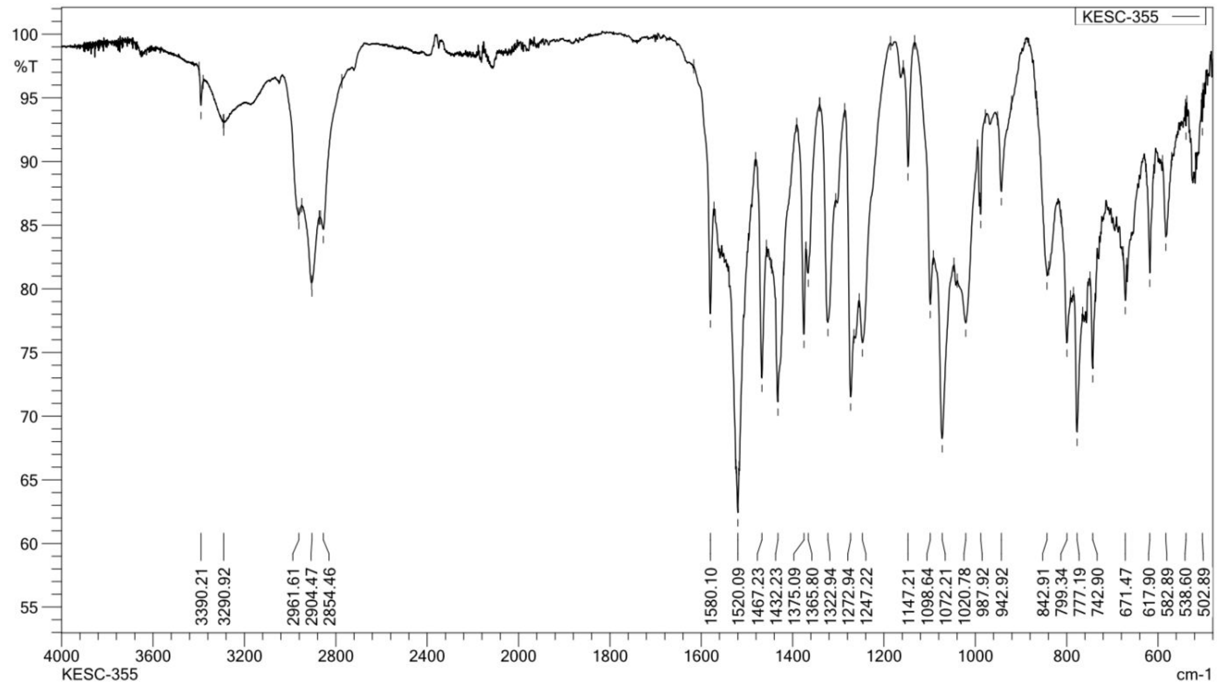

Figure S13: IR Spectrum of **Ti1a**.

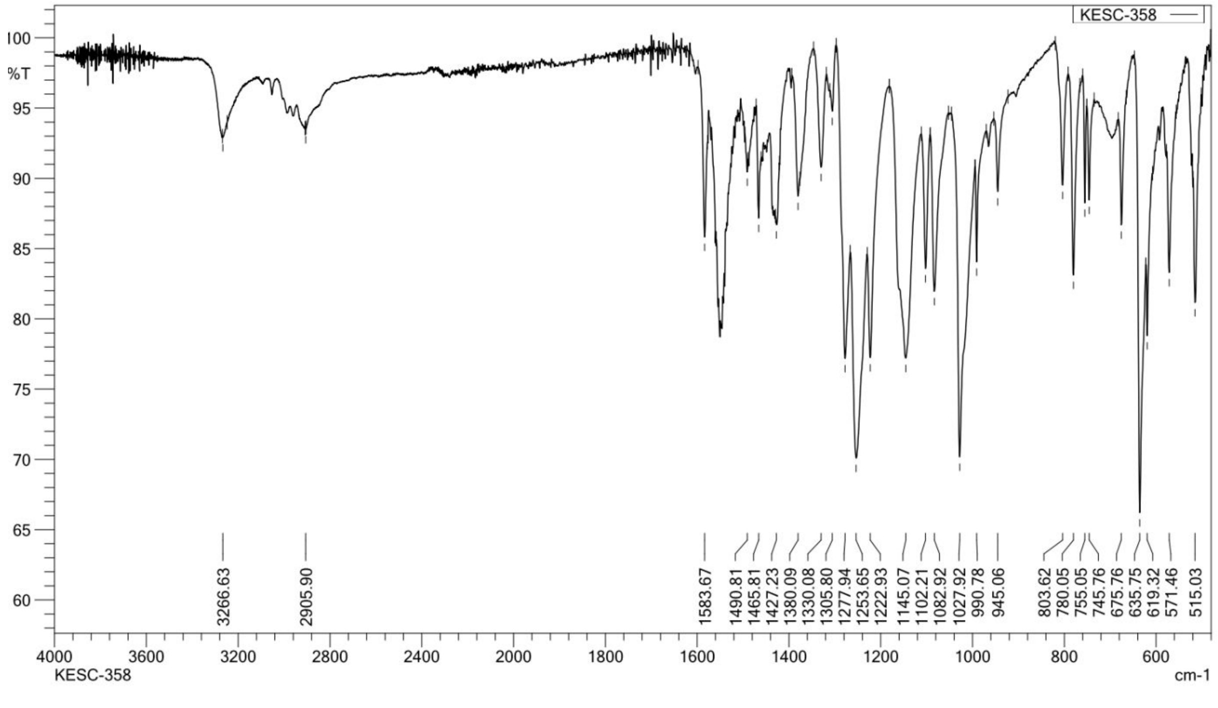

Figure S14: IR Spectrum of **Ti2a**.

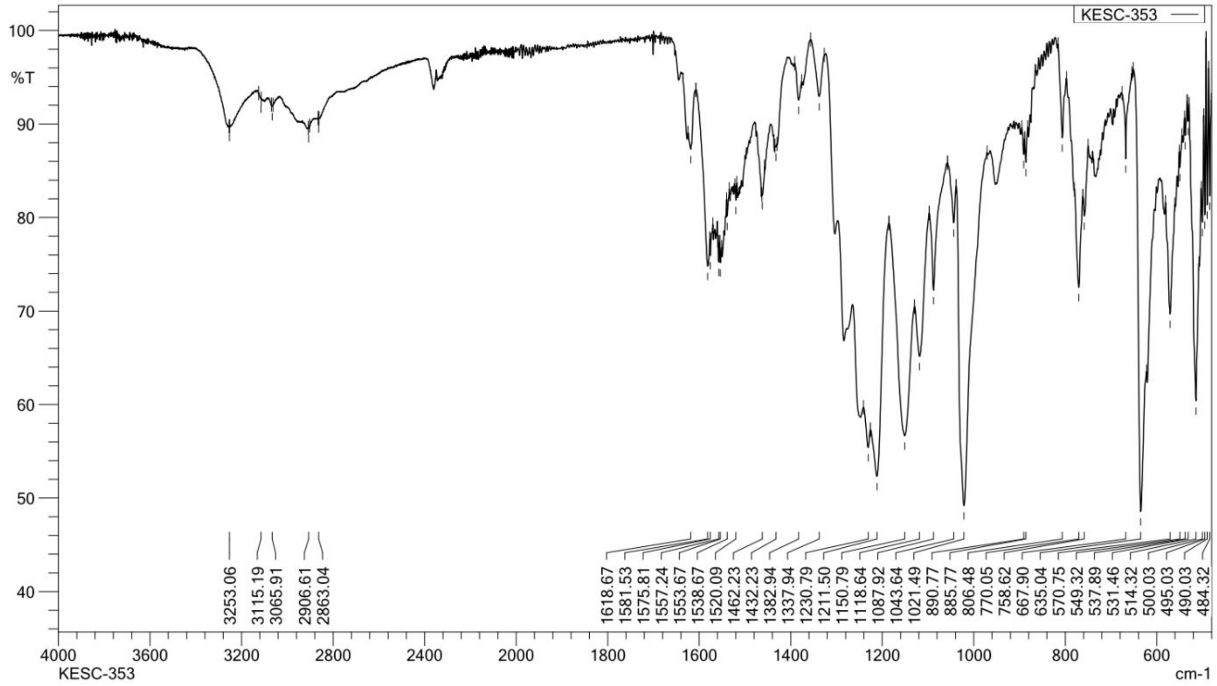

Figure S15: IR Spectrum of **Ti3a**.

Computational details

Computational methods

The DFT (density functional theory) calculations were performed with the B3LYP/Def2-TZVP level of theory.[5] The optimized ground state geometries for the mechanistic studies of the pyridinium ion formation (**A**, **B**, **Ti3a**, **TS1**, **TS2**) were calculated in THF at room temperature using the SMD solvation model.[6] Similarly, the optimized ground state geometries for the mechanistic studies of the hydrolyzation reactions were calculated in water at room temperature using the SMD solvation model.[6]

Table S2: Optimized ground state geometries (calculated in THF) of **A**, **TS1**, **B**, **TS2**, **Ti3a^{2+}** and **Ti3a^{2+}NNS**:

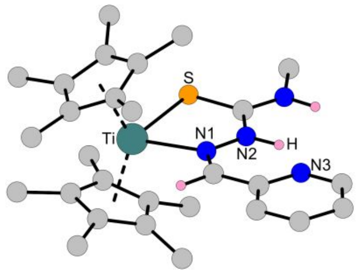

A
Ti–S: 2.56 Å
Ti–N1: 2.26 Å
N2–H: 1.04 Å
N3–H: 1.76 Å

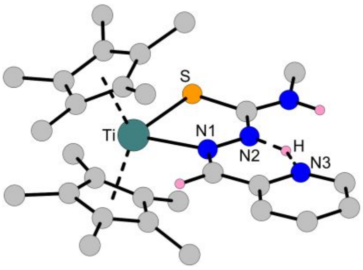

TS1
Ti–S: 2.54 Å
Ti–N1: 2.24 Å
N2–H: 1.28 Å
N3–H: 1.27 Å

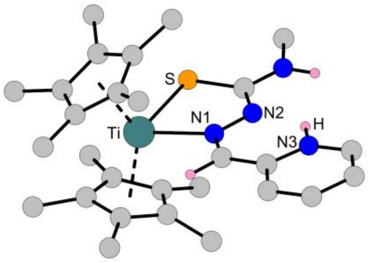

B
Ti–S: 2.52 Å
Ti–N1: 2.24 Å
N2–H: 1.85 Å
N3–H: 1.03 Å

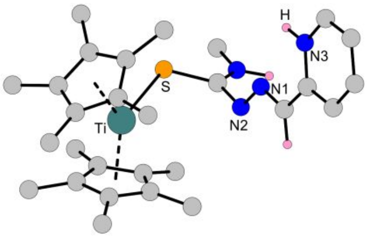

TS2
Ti–S: 2.52 Å
Ti–N1: 4.43 Å
Ti–N2: 3.81 Å

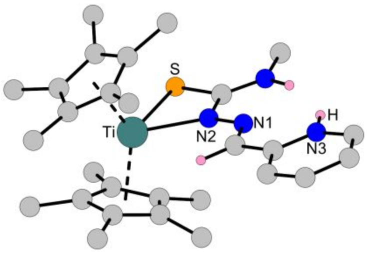

Ti3a^{2+}
Ti–S: 2.57 Å
Ti–N2: 2.22 Å

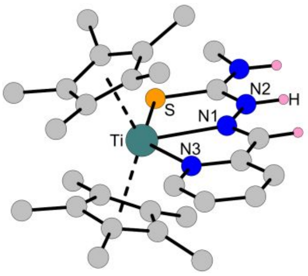

Ti3a^{2+}NNS
Ti–S: 2.70 Å
Ti–N1: 2.36 Å
Ti–N3: 2.37Å

Table S3: Calculated Gibbs free energies at 298 K (G(298)) in THF at the B3LYP/Def2-TZVP level of theory:

Compound	G(298) [kJ/mol]	ΔG(298) [kJ/mol]
**A**	-69565.9926460	0
**TS1**	-69565.8979247	9.139101111
**B**	-69566.0122123	-1.887938737
**TS2**	-69564.8445132	110.7775122
**Ti3a^{2+}**	-69566.1220783	-12.4883624
**Ti3a^{2+}NNS**	-69565.5657609	41.18792973

Hydrolyzation of Cp-based complex

Table S4: Optimized ground state geometries (calculated in H_2O) of **Cp-TSCN**, **TS1$_N$**, **TS1$_S$**, **Cp-N**, **Cp-S**, **TS2$_N$**, **TS2$_S$** and **Cp-aq**:

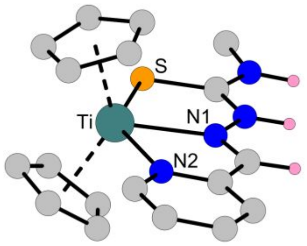

<u>**Cp-TSCN**</u>
Ti–S: 2.71 Å
Ti–N1: 2.32 Å
Ti–N2: 2.38 Å

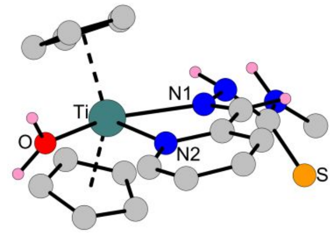

<u>**TS1$_N$**</u>
Ti–O: 2.28 Å
Ti–N1: 2.35 Å
Ti–N2: 2.34 Å

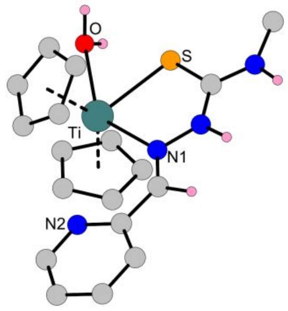

<u>**TS1$_S$**</u>
Ti–O: 2.68 Å
Ti–S: 2.51 Å
Ti–N1: 2.47 Å

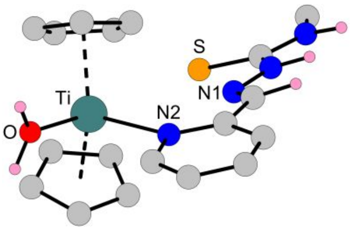

<u>**Cp-N**</u>
Ti–O: 2.09 Å
Ti···N1: 3.70 Å
Ti–N2: 2.29 Å

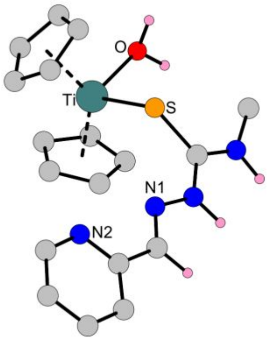

<u>**Cp-S**</u>
Ti–O: 2.12 Å
Ti–S: 2.46 Å
Ti···N1: 4.59 Å

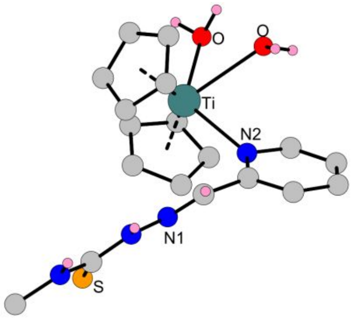

<u>**TS2$_N$**</u>
Ti–O1: 2.19 Å
Ti–O2: 2.64 Å
Ti–N2: 2.47 Å

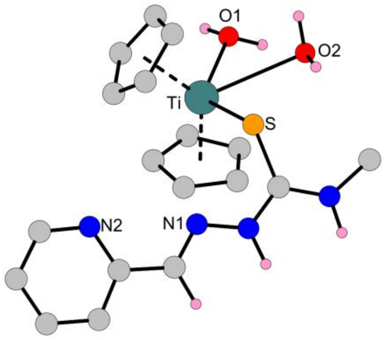

<u>**TS2$_S$**</u>
Ti–O1: 2.22 Å
Ti–O2: 2.89 Å
Ti–S: 2.66 Å

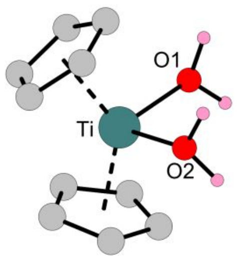

<u>**Cp-aq**</u>
Ti–O1: 2.08 Å
Ti–O2: 2.09 Å

Table S5: Calculated Gibbs free energies at 298 K (G(298)) in water at the B3LYP/Def2-TZVP level of theory:

**Compound**	**G(298) [kJ/mol]**	**ΔG(298) [kJ/mol]**
**Cp-TSCN** + 2 H_2O	-6082316.593	0
**$TS1_N$** + H_2O	-6082212.082	104.5105943
**$TS1_S$** + H_2O	-6082182.938	133.6550775
**Cp-N** + H_2O	-6082214.740	101.8521524
**Cp-S** + H_2O	-6082253.772	62.82051295
**$TS2_N$**	-6082101.303	215.2897081
**$TS2_S$**	-6082143.780	172.8125043
**Cp-aq** + TSCN	-6082266.365	50.22755096
**Cp-TSCN**	-5680953.210	
**$TS1_N$**	-5881530.391	
**$TS1_S$**	-5881501.246	
**Cp-N**	-5881533.049	
**Cp-S**	-5881572.081	
**Cp-aq**	-3646420.549	
H_2O	-200681.691	
TSCN	-2435845.816	

Hydrolyzation of Cp*-based complex

Table S6: Optimized ground state geometries (calculated in H_2O) of **Ti3a^{2+}**, **TS1$_{N}$***, **TS1$_{S}$***, **Cp*-N**, **Cp*-S**, **Cp*-N2**, **TS2$_{N}$***, **TS2$_{S}$***and **Cp*-aq**:

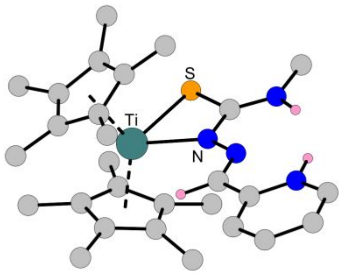

Ti3a^{2+}

Ti–S: 2.58 Å

Ti–N: 2.20 Å

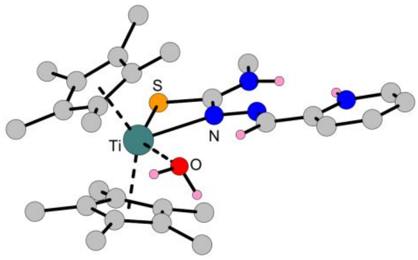

TS1$_{N}$*

Ti–S: 2.60 Å

Ti–N: 2.31 Å

Ti–O: 2.90 Å

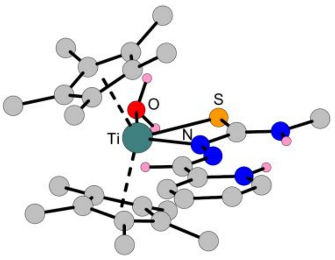

TS1$_{S}$*

Ti–O: 2.74 Å

Ti–S: 2.57 Å

Ti–N: 2.30 Å

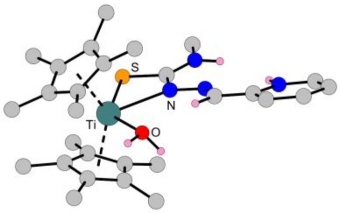

Cp*-N

Ti–S: 2.63 Å

Ti–N: 2.40 Å

Ti–O: 2.23 Å

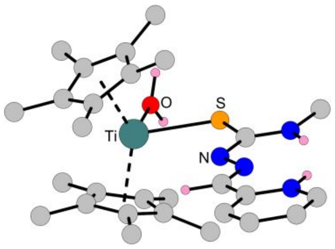

Cp*-S

Ti–O: 2.10 Å

Ti–S: 2.57 Å

Ti···N: 3.76 Å

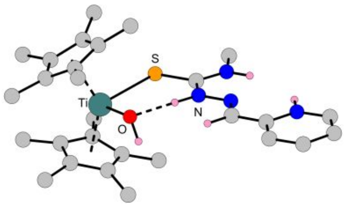

Cp*-N2

Ti–S: 2.68 Å

Ti–O: 1.91 Å

N–H: 1.03 Å

O···H: 1.78 Å

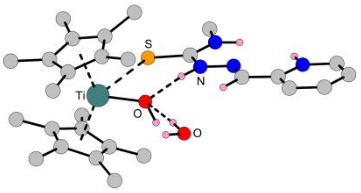

TS2$_{N}$*

Ti···S: 3.67 Å

Ti–O: 1.91 Å

O1···HN: 1.97 Å

O1···HO2: 2.14 Å

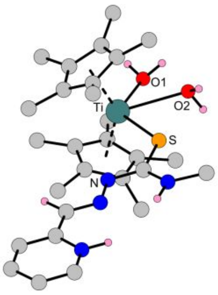

TS2$_{S}$*

Ti–O1: 2.23 Å

Ti–O2: 2.67 Å

Ti–S: 2.70 Å

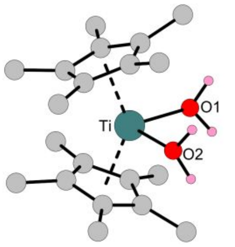

Cp*-aq

Ti–O1: 2.11 Å

Ti–O2: 2.10 Å

Table S7: Calculated Gibbs free energies at 298 K (G(298)) in water at the B3LYP/Def2-TZVP level of theory:

**Compound**	**G(298) [kJ/mol]**	**ΔG(298) [kJ/mol]**
**Ti3a**$^{2+}$ + 2 H_2O	-7113466.985	0
**TS1**$_N$***** + H_2O	-7113352.841	114.1440755
**TS1**$_S$***** + H_2O	-7113365.266	101.7187774
**Cp*-N** + H_2O	-7113359.959	107.0258943
**Cp*-S** + H_2O	-7113382.464	84.52143773
**Cp*-N2** + H_2O	-7113446.960	20.02523081
**TS2**$_N$*****	-7113391.639	75.34573726
**TS2**$_S$*****	-7113247.370	219.6149697
**Cp-aq*** + TSCN	-7113429.283	37.70188032
**Ti3a**$^{2+}$	-6712103.602	
**TS1**$_N$*****	-6912671.150	
**TS1**$_S$*****	-6912683.575	
**Cp*-N**	-6912678.268	
**Cp*-S**	-6912700.772	
**Cp*-N2**	-6912765.269	
**Cp-aq***	-4677583.467	
H_2O	-200681.691	
TSCN	-2435845.816	

Table S8: Cartesian coordinates (Å) of **A**, **TS1**, **B**, **TS2**, **Ti3a^{2+}** and **Ti3a^{2+}NNS**:

A				TS1			
Ti	5.850996	2.755470	2.257714	Ti	5.821908	2.723269	2.236540
N	4.503324	2.457001	4.048549	N	4.603633	2.491047	4.105344
C	8.195549	2.139179	2.553242	C	8.144146	2.070165	2.571086
C	4.410098	4.608063	1.500264	C	4.402101	4.529450	1.328752
C	5.417283	4.381528	0.512483	C	5.514086	4.340352	0.452648
C	7.806697	1.675156	1.258740	C	7.813305	1.706036	1.230396
C	6.599809	0.497087	2.831289	C	6.545903	0.409761	2.654354
C	6.827808	0.660897	1.434031	C	6.811642	0.700041	1.283348
C	6.458815	5.090277	2.441989	C	6.321930	5.079020	2.479419
C	7.452284	1.406067	3.517275	C	7.369895	1.254220	3.442851
C	5.053921	5.035632	2.687141	C	4.899681	4.973044	2.577924
C	6.671625	4.746851	1.074879	C	6.687691	4.758010	1.142336
S	3.830661	1.506121	1.301725	S	3.747214	1.471143	1.465060
C	2.837473	1.461132	2.681884	C	2.863620	1.479672	2.930727
C	4.835685	2.819089	5.246633	C	5.026721	2.922253	5.257566
N	3.256016	1.936527	3.873342	N	3.353901	1.961134	4.084532
H	2.686118	1.873906	4.742876	H	2.871103	2.011678	5.264760
N	1.614665	0.945829	2.650793	N	1.632296	0.974928	2.984805
C	0.974188	0.375738	1.474076	C	0.909782	0.386380	1.868851
H	1.090447	0.926821	3.516681	H	1.174661	0.977475	3.886430
H	0.909298	1.110577	0.670684	H	0.806913	1.098878	1.049065
H	-0.030169	0.074039	1.760438	H	-0.080654	0.111821	2.223727
H	1.519519	-0.499160	1.116263	H	1.413632	-0.508924	1.499380
C	4.371171	5.595527	3.891446	C	4.067406	5.507149	3.697938
H	4.179166	6.661560	3.725254	H	3.785518	6.541048	3.469747
H	3.408379	5.123609	4.085135	H	3.142338	4.947859	3.837697
H	4.986318	5.521975	4.787596	H	4.610784	5.528682	4.641479
C	7.449895	5.725641	3.367435	C	7.206729	5.713070	3.508340
H	7.265186	6.804328	3.413791	H	7.062586	6.799098	3.499960
H	7.370051	5.347086	4.387447	H	6.980716	5.371922	4.519488
H	8.473252	5.585898	3.029555	H	8.259968	5.525391	3.312815
C	7.890188	5.073249	0.274145	C	7.970449	5.158177	0.489126
H	7.821224	6.128402	-0.015656	H	7.904685	6.230295	0.268895
H	8.817465	4.959021	0.828431	H	8.842404	5.023723	1.123765
H	7.952507	4.495896	-0.644057	H	8.139104	4.651381	-0.456539
C	5.172616	4.041025	-0.924759	C	5.406687	4.015128	-1.005075
H	5.066660	4.957852	-1.514846	H	5.116693	4.910299	-1.566137
H	5.997120	3.473703	-1.356366	H	6.350222	3.665442	-1.419888
H	4.256959	3.465653	-1.058333	H	4.645870	3.258542	-1.201745
C	2.936401	4.658166	1.262162	C	2.967369	4.549017	0.914567
H	2.655053	5.690597	1.025224	H	2.718817	5.559668	0.569847
H	2.626846	4.037991	0.423832	H	2.760999	3.865717	0.093328
H	2.362201	4.367759	2.142021	H	2.291718	4.314865	1.736241
C	5.781476	-0.577667	3.470328	C	5.725797	-0.722013	3.181872
H	6.412048	-1.452961	3.659898	H	6.380455	-1.575512	3.389868
H	5.369413	-0.267521	4.430638	H	5.225835	-0.470355	4.117955
H	4.961958	-0.904965	2.832084	H	4.975567	-1.055111	2.467860
C	6.342372	-0.234541	0.338804	C	6.324881	-0.069726	0.095991
H	7.140098	-0.939036	0.078298	H	7.084680	-0.802604	-0.196923

H	5.474591	-0.820184	0.633499	H	5.408421	-0.618453	0.303558
H	6.095425	0.314213	-0.571306	H	6.152819	0.572941	-0.768559
C	8.527451	1.903451	-0.032890	C	8.603279	2.013486	-0.002609
H	9.084201	0.994504	-0.285298	H	9.195931	1.128380	-0.259252
H	7.852765	2.099531	-0.866751	H	7.976470	2.231272	-0.866827
H	9.246639	2.714815	0.029274	H	9.298330	2.834812	0.140942
C	9.412151	2.945625	2.879794	C	9.330017	2.866772	3.017147
H	10.252922	2.260825	3.038920	H	10.155694	2.181349	3.239122
H	9.699210	3.621412	2.079438	H	9.684907	3.555724	2.255966
H	9.294031	3.521711	3.795737	H	9.133559	3.430534	3.928489
C	7.720570	1.412444	4.988503	C	7.591327	1.122267	4.914837
H	7.958566	2.406106	5.368547	H	7.899940	2.059291	5.377751
H	6.891395	1.005850	5.565016	H	6.713950	0.744543	5.437742
H	8.591245	0.778910	5.191843	H	8.400015	0.402649	5.086708
H	5.821985	3.251143	5.332264	H	6.011215	3.364350	5.275733
C	4.069496	2.733134	6.485828	C	4.293937	2.873984	6.510024
C	4.662339	3.181298	7.669461	C	4.831593	3.351568	7.704666
C	3.939409	3.101143	8.850892	C	4.065194	3.272298	8.857754
H	5.667510	3.581487	7.655030	H	5.827547	3.772914	7.718043
C	2.134904	2.154526	7.597665	C	2.307423	2.265501	7.584318
C	2.653781	2.579048	8.817974	C	2.786694	2.723735	8.801529
H	4.374000	3.441564	9.781839	H	4.460540	3.636907	9.796869
H	1.134319	1.743105	7.534067	H	1.322485	1.830206	7.477790
H	2.056092	2.499018	9.715926	H	2.168081	2.650813	9.685020
N	2.819909	2.227942	6.461098	N	3.050280	2.344847	6.481735

**B**				**TS2**			
Ti	5.801984	2.726039	2.230711	Ti	0.491851	9.362490	3.412072
N	4.480642	2.446359	4.019996	N	4.068268	8.484677	4.376687
C	8.140930	2.092890	2.580275	C	-0.751846	7.490864	4.169909
C	4.388069	4.572583	1.385003	C	0.858951	11.112674	1.838815
C	5.474242	4.375997	0.479958	C	-1.788261	9.546815	4.186938
C	7.794645	1.679696	1.257008	C	-0.196053	10.331570	1.297313
C	6.540591	0.440799	2.743026	C	2.049724	10.316608	1.844755
C	6.805387	0.666419	1.360352	C	-0.320230	8.192593	5.339305
C	6.345627	5.070241	2.496665	C	-1.711635	8.298143	3.501116
C	7.372938	1.317233	3.490325	C	0.318507	9.025536	1.033467
C	4.925537	4.991906	2.626574	C	-0.951885	9.465844	5.339688
C	6.672757	4.756590	1.146541	C	1.701514	9.022492	1.375961
S	3.789416	1.500181	1.323179	S	2.130687	10.240722	5.110851
C	2.846813	1.467003	2.759495	C	3.761738	9.681519	4.962608
C	4.900450	2.866853	5.185041	N	4.080051	7.430426	5.134987
N	3.252983	1.915729	3.958065	C	4.397938	6.270811	4.601198
N	1.618336	0.944395	2.735537	N	4.753580	10.428729	5.399127
C	0.955505	0.371118	1.578388	C	4.673260	11.784912	5.924214
H	1.127100	0.923654	3.618624	H	5.667606	9.989895	5.380757
H	0.869885	1.100499	0.770887	N	4.180964	5.241070	6.769523
H	-0.042773	0.065980	1.883573	C	4.211264	4.214675	7.645003
H	1.492835	-0.503811	1.205863	C	4.450533	5.124219	5.439483

C	4.131529	5.536944	3.768573	H	3.951738	6.171677	7.105800
H	3.873237	6.579975	3.553311	C	3.431334	10.857727	2.017496
H	3.194388	5.002087	3.922694	C	0.776457	12.564652	2.189727
H	4.693841	5.535953	4.701020	C	-1.495297	10.893776	0.819399
C	7.257325	5.681035	3.516787	C	-0.333696	7.943782	0.230959
H	7.068565	6.757951	3.583014	C	2.621288	7.862929	1.159534
H	7.098419	5.270334	4.515341	H	3.480560	11.634909	2.778665
H	8.305255	5.550774	3.257943	H	3.744446	11.315066	1.072531
C	7.943851	5.130494	0.455429	H	4.153947	10.082294	2.259995
H	7.879576	6.195570	0.202871	H	0.898083	13.173637	1.287168
H	8.827980	5.009401	1.075078	H	1.557328	12.859261	2.889481
H	8.088357	4.593188	-0.477675	H	-0.188973	12.825152	2.624700
C	5.340029	4.057226	-0.976743	H	-1.844919	11.715171	1.441696
H	5.195397	4.978072	-1.552420	H	-2.282485	10.148502	0.749244
H	6.227707	3.565452	-1.372455	H	-1.343827	11.298361	-0.187745
H	4.480166	3.417156	-1.175542	H	-1.398879	8.110724	0.100173
C	2.940147	4.614506	1.021749	H	-0.185609	6.953119	0.661660
H	2.681069	5.640232	0.734373	H	0.117916	7.926503	-0.766768
H	2.701416	3.969909	0.178690	H	3.615847	8.059137	1.552397
H	2.294582	4.343603	1.856842	H	2.713173	7.657238	0.088110
C	5.711981	-0.663824	3.313819	H	2.243288	6.949737	1.624613
H	6.342288	-1.544085	3.481388	C	0.498501	7.586520	6.431906
H	5.274140	-0.397048	4.275855	C	-0.907708	10.494493	6.424302
H	4.910209	-0.964198	2.641359	C	-2.786261	10.637367	3.952140
C	6.338784	-0.175693	0.215146	C	-2.689782	7.803552	2.486971
H	7.122819	-0.896931	-0.041308	C	-0.375618	6.082210	3.831404
H	5.441384	-0.741839	0.454783	H	1.363950	7.047847	6.045613
H	6.144093	0.413629	-0.681994	H	-0.116782	6.863596	6.978611
C	8.563297	1.952275	0.001871	H	0.848722	8.329800	7.144264
H	9.144134	1.058080	-0.249792	H	-0.037514	10.384102	7.068243
H	7.921126	2.158542	-0.854495	H	-1.796587	10.390851	7.055973
H	9.267205	2.771571	0.113180	H	-0.916883	11.510052	6.026132
C	9.340869	2.892150	2.980707	H	-2.339234	11.630493	4.015544
H	10.169597	2.205617	3.188064	H	-3.561755	10.588055	4.723807
H	9.677625	3.568263	2.200362	H	-3.279594	10.541993	2.988400
H	9.172244	3.468338	3.889274	H	-3.012365	8.571514	1.789400
C	7.611168	1.247699	4.964868	H	-3.583455	7.467100	3.025148
H	7.883011	2.212815	5.392330	H	-2.313957	6.951261	1.927205
H	6.753879	0.853706	5.507943	H	-0.528152	5.859933	2.776089
H	8.450473	0.569041	5.155213	H	-0.989443	5.380182	4.406105
H	5.882346	3.314099	5.204273	H	0.665916	5.872466	4.079551
C	4.219415	2.821463	6.454875	H	4.050838	12.403579	5.278768
C	4.826107	3.304508	7.618269	H	5.680808	12.192934	5.955647
C	4.151102	3.241774	8.823548	H	4.253654	11.788038	6.931777
H	5.821649	3.721108	7.556824	H	4.647716	6.161082	3.549772
C	2.293211	2.233084	7.720388	C	4.779682	3.834936	4.976980
C	2.865479	2.698802	8.881893	C	4.816640	2.773209	5.849935
H	4.621761	3.615692	9.723405	H	5.002252	3.704427	3.926985
H	1.304836	1.799700	7.670242	C	4.527530	2.955372	7.214441
H	2.318187	2.639457	9.811287	H	5.071601	1.787040	5.483187
H	2.573750	1.964488	5.674403	H	4.553007	2.133736	7.914599
N	2.969485	2.305515	6.564186	H	3.976609	4.460372	8.670600

Ti3a^{2+}				Ti3a^{2+}NNS			
Ti	0.691240	9.368444	3.611832	Ti	6.079120	2.958151	2.599904
N	2.533240	8.714360	4.653887	N	4.405838	2.466268	4.196615
C	-0.667516	7.724015	4.818751	N	6.686946	3.676536	4.773369
C	0.941994	10.846946	1.698143	C	8.397944	2.116004	2.433242
C	-1.633790	9.733014	4.246088	C	4.586352	4.788212	1.835656
C	-0.017147	9.870042	1.318521	C	5.356329	4.393148	0.695907
C	2.200960	10.193658	1.846563	C	7.733639	1.661040	1.247413
C	-0.252413	8.696693	5.772819	C	6.771340	0.600973	3.048504
C	-1.535490	8.355362	3.889190	C	6.769957	0.699602	1.626639
C	0.628161	8.596652	1.312429	C	6.782613	5.371875	2.213197
C	-0.862301	9.933518	5.424711	C	7.837665	1.407388	3.532612
C	2.002739	8.809465	1.620862	C	5.466826	5.383679	2.770746
S	1.976970	11.206599	4.872352	C	6.702104	4.781702	0.928273
C	2.985881	9.866776	5.210786	S	3.988829	1.713157	1.421128
N	3.263467	7.631691	4.932550	C	2.921383	1.417331	2.687369
C	2.919642	6.484009	4.463403	C	4.581976	2.816375	5.421001
N	4.086661	9.936008	5.943581	N	3.238259	1.825811	3.938150
C	4.605200	11.147940	6.557710	H	2.602981	1.659289	4.714425
H	4.592299	9.070789	6.074764	N	1.757217	0.794479	2.538323
N	4.811563	5.461780	5.570659	C	1.243669	0.291045	1.272433
C	5.617418	4.447582	5.922567	H	1.176030	0.661865	3.355783
C	3.721363	5.321867	4.778862	C	7.837082	4.276075	5.089820
H	5.018993	6.397733	5.911648	C	5.802985	3.483036	5.777269
C	3.536117	10.849790	1.973475	H	1.111633	1.101399	0.554034
C	0.702719	12.323900	1.738738	H	0.278770	-0.169960	1.468601
C	-1.330281	10.200735	0.683815	H	1.915668	-0.455833	0.847434
C	0.081243	7.318789	0.752803	H	3.837838	2.615743	6.186833
C	3.095932	7.802320	1.454158	C	6.036021	3.889977	7.087978
H	3.469134	11.857182	2.377785	C	7.227758	4.528298	7.391988
H	3.991050	10.923991	0.979107	H	5.284152	3.703117	7.842869
H	4.224297	10.274108	2.593351	C	8.143811	4.721025	6.370246
H	0.743001	12.740500	0.726480	H	7.436577	4.860605	8.400323
H	1.454626	12.842571	2.332147	H	9.095486	5.203566	6.546134
H	-0.279132	12.566601	2.147141	H	8.551487	4.401966	4.295757
H	-1.786465	11.092763	1.106370	C	5.021615	6.143057	3.978424
H	-2.048620	9.386751	0.723042	H	4.639728	7.121762	3.666800
H	-1.148294	10.411550	-0.376697	H	4.207783	5.643388	4.505161
H	-0.990526	7.375630	0.582190	H	5.830510	6.326369	4.682946
H	0.278456	6.456809	1.392404	C	7.932490	6.220791	2.660467
H	0.554574	7.112542	-0.212976	H	7.955818	7.126713	2.043707
H	3.926371	7.970486	2.138883	H	7.838370	6.545480	3.694204
H	3.498312	7.882341	0.437676	H	8.899477	5.733803	2.534729
H	2.741867	6.780285	1.578022	C	7.737126	4.947617	-0.136365
C	0.501296	8.429163	7.035745	H	7.667221	5.976092	-0.511338
C	-0.926157	11.162417	6.274124	H	8.754458	4.814902	0.226220
C	-2.618205	10.731199	3.718207	H	7.575845	4.288609	-0.984464
C	-2.451746	7.607139	2.974438	C	4.826913	3.986765	-0.645491
C	-0.457449	6.243875	4.907534	H	4.844502	4.851960	-1.317325
H	1.167886	7.572666	6.946483	H	5.431565	3.209587	-1.114671

H	-0.203598	8.210014	7.845098	H	3.798704	3.636686	-0.598109
H	1.088500	9.290775	7.353254	C	3.094892	4.862530	1.930672
H	-0.089794	11.232990	6.965864	H	2.775006	5.889382	1.720828
H	-1.845578	11.134146	6.870245	H	2.599144	4.216160	1.211029
H	-0.958514	12.075938	5.679577	H	2.726755	4.617469	2.927933
H	-2.163403	11.700251	3.506296	C	6.021577	-0.413596	3.853511
H	-3.395285	10.903649	4.470354	H	6.586623	-1.351235	3.896762
H	-3.112760	10.380531	2.816745	H	5.864394	-0.087759	4.881943
H	-2.832848	8.213999	2.158285	H	5.051350	-0.646897	3.415737
H	-3.319897	7.274726	3.555777	C	6.159693	-0.285413	0.682775
H	-1.989639	6.714096	2.558632	H	6.925523	-1.032080	0.438902
H	-0.253985	5.790975	3.936242	H	5.313458	-0.814242	1.113865
H	-1.368697	5.770923	5.290337	H	5.844670	0.164573	-0.258294
H	0.350855	5.982902	5.587772	C	8.245557	1.795197	-0.152490
H	4.843911	11.899638	5.803303	H	8.736306	0.856255	-0.433075
H	5.514218	10.887650	7.094993	H	7.453321	1.966147	-0.881348
H	3.887090	11.570441	7.262777	H	8.983587	2.585069	-0.250196
H	2.054586	6.333256	3.828018	C	9.706412	2.849061	2.430856
C	3.427714	4.045051	4.304846	H	10.485930	2.196049	2.023423
C	4.240064	2.977623	4.650473	H	9.691342	3.745792	1.814296
H	2.562759	3.908547	3.671188	H	10.026065	3.131993	3.431902
C	5.351972	3.175387	5.470179	C	8.424610	1.256279	4.898584
H	4.010124	1.986461	4.281733	H	9.155531	2.026099	5.133840
H	6.000646	2.359635	5.753329	H	7.670686	1.237268	5.685580
H	6.453004	4.698099	6.559674	H	8.948271	0.294681	4.940626

Table S9: Cartesian coordinates (Å) of **Cp-TSCN**, **TS1$_N$**, **TS1$_S$**, **Cp-N**, **Cp-S**, **TS2$_N$**, **TS2$_S$** and **Cp-aq**:

**Cp-TSCN**				**TS1$_N$**			
Ti	6.086720	2.971522	2.546796	Ti	7.022998	3.290578	2.597450
N	4.472563	2.491787	4.142267	N	5.157760	2.305698	3.634855
N	6.756371	3.733858	4.694495	N	6.795481	4.060372	4.798761
C	8.352693	2.256250	2.489811	C	9.002850	1.960272	3.043096
C	4.670156	4.786629	1.811764	C	5.230272	4.835021	2.102032
C	5.366084	4.312785	0.665187	C	5.120994	3.782561	1.157700
C	7.813725	1.893722	1.230814	C	8.884856	2.142962	1.640145
C	6.754765	0.657434	2.839138	C	7.154800	0.834205	2.335843
C	6.829650	0.916661	1.444316	C	7.754618	1.414571	1.206605
C	6.900146	5.188735	2.103851	C	7.094426	4.853643	0.778899
C	7.724631	1.453365	3.473708	C	7.923044	1.182120	3.478452
C	5.614377	5.338272	2.689812	C	6.441968	5.501576	1.861241
C	6.733695	4.589551	0.839797	C	6.261846	3.807828	0.334698
S	3.990785	1.740893	1.352828	S	2.095740	2.931175	3.989869
C	2.966726	1.426139	2.660136	C	2.837077	1.744497	3.066430
C	4.664867	2.851799	5.361687	C	4.993634	2.507740	4.890481
N	3.307615	1.836706	3.900526	N	4.215415	1.504077	3.030070
H	2.687141	1.665587	4.688576	H	4.556329	1.105452	2.163778
N	1.812475	0.784993	2.537941	N	2.167188	0.909962	2.266231
C	1.276567	0.292739	1.276498	C	0.728747	0.938213	2.061913
H	1.249974	0.646318	3.367691	H	2.687443	0.218276	1.741647
C	7.910174	4.338371	4.988049	C	7.522736	5.016967	5.385438
C	5.884647	3.529307	5.704013	C	5.851567	3.453363	5.557206
H	3.607149	4.725828	1.979526	H	4.515638	5.072729	2.873190
H	4.924636	3.853168	-0.203472	H	4.292662	3.103988	1.053658
H	7.516927	4.381439	0.131759	H	6.454590	3.154863	-0.498532
H	7.834613	5.524819	2.518994	H	8.047494	5.121504	0.352991
H	5.400854	5.778095	3.650152	H	6.825908	6.334707	2.428435
H	6.087266	-0.038860	3.321447	H	6.301713	0.178377	2.327214
H	6.251334	0.432147	0.677083	H	7.420851	1.302347	0.189438
H	8.116933	2.279008	0.272730	H	9.551571	2.707874	1.009724
H	9.145954	2.967282	2.651010	H	9.770445	2.370193	3.680227
H	7.932524	1.463335	4.529957	H	7.713430	0.896904	4.497155
H	1.095104	1.113340	0.581643	H	0.199678	0.818163	3.006660
H	0.334653	-0.204096	1.491587	H	0.474948	0.112712	1.401013
H	1.958843	-0.422257	0.816479	H	0.411810	1.873873	1.600613
H	3.933150	2.646975	6.137565	H	4.237800	1.986015	5.460115
C	6.135035	3.929392	7.012481	C	5.653701	3.744105	6.900538
C	7.332846	4.564281	7.301441	C	6.432959	4.722703	7.499766
H	5.394248	3.735910	7.775891	H	4.886134	3.213913	7.447279
C	8.234529	4.767972	6.268901	C	7.370423	5.377272	6.720105
H	7.557218	4.887147	8.309031	H	6.298917	4.973847	8.543496
H	9.186929	5.251325	6.435343	H	7.990102	6.166253	7.123003
H	8.613657	4.486549	4.184151	H	8.247190	5.521005	4.769789
				O	8.809807	4.664249	2.908711
				H	9.647183	4.402694	3.313170
				H	8.988335	5.465489	2.400207

TS1$_S$				Cp-N			
Ti	5.803101	2.489931	1.726958	Ti	7.426285	3.667929	2.361643
N	4.660603	2.622966	3.911679	N	4.302647	2.452241	3.927428
N	7.247508	3.936468	4.833397	N	6.919146	3.980317	4.575018
C	8.023440	1.949802	2.413169	C	8.695939	1.696703	2.870288
C	4.643986	4.608226	1.416659	C	5.410715	4.310804	1.337217
C	5.186486	4.250320	0.171074	C	6.424280	4.441521	0.350091
C	7.824195	1.404904	1.120152	C	8.811639	1.908769	1.475877
C	6.445751	0.298946	2.573293	C	6.611588	1.492696	1.949532
C	6.874075	0.362478	1.236320	C	7.530096	1.783792	0.906847
C	6.924185	4.599228	1.615575	C	6.843237	5.965377	2.001174
C	7.172331	1.269562	3.303643	C	7.342597	1.417086	3.155798
C	5.721536	4.839229	2.305380	C	5.660983	5.271556	2.340192
C	6.596455	4.208958	0.293663	C	7.305090	5.459320	0.762296
S	3.556390	1.430570	1.315859	S	2.830302	1.719564	1.265634
C	2.789357	1.536266	2.830088	C	2.478873	1.281632	2.849364
C	4.962506	3.078205	5.085712	C	4.827375	2.652264	5.080568
N	3.384458	2.097036	3.895758	N	3.216390	1.652622	3.948400
H	2.872909	2.128877	4.774200	H	2.913343	1.301436	4.856066
N	1.561622	1.067162	3.016521	N	1.430506	0.512687	3.175148
C	0.747392	0.447625	1.979032	C	0.488227	-0.048207	2.223852
H	1.152839	1.147864	3.938496	H	1.274199	0.295819	4.150037
C	8.307058	4.510442	5.390347	C	7.856786	4.798826	5.091783
C	6.172988	3.708294	5.602657	C	5.972359	3.496698	5.427670
H	3.595702	4.697569	1.652131	H	4.590409	3.608887	1.321600
H	4.614858	4.031603	-0.715136	H	6.494968	3.879850	-0.565433
H	7.295653	3.958107	-0.486872	H	8.199356	5.769557	0.244479
H	7.915293	4.707023	2.019710	H	7.322725	6.731483	2.591007
H	5.632215	5.154836	3.328113	H	5.069175	5.424770	3.227216
H	5.703316	-0.372484	2.973663	H	5.549940	1.344126	1.830685
H	6.518452	-0.267342	0.438804	H	7.293398	1.866946	-0.139798
H	8.329164	1.707520	0.217681	H	9.714592	2.169399	0.946516
H	8.713654	2.731582	2.675520	H	9.496904	1.756444	3.591521
H	7.091988	1.446498	4.359942	H	6.941222	1.195833	4.130064
H	0.571187	1.139719	1.155438	H	-0.028881	0.735701	1.669678
H	-0.206068	0.182269	2.427177	H	-0.241021	-0.626556	2.786616
H	1.222165	-0.455477	1.594951	H	0.989378	-0.704009	1.511117
H	4.196550	2.985275	5.852867	H	4.369008	2.200573	5.961316
C	6.139029	4.055830	6.959427	C	6.033967	3.797060	6.795578
C	7.253505	4.654389	7.529503	C	7.016105	4.622106	7.307229
H	5.254406	3.858204	7.549912	H	5.281414	3.378307	7.448512
C	8.362266	4.888116	6.731739	C	7.946026	5.146099	6.426297
H	7.251407	4.931240	8.575543	H	7.047960	4.851548	8.363959
H	9.255391	5.352112	7.128154	H	8.738530	5.806610	6.748136
H	9.160333	4.681719	4.742500	H	8.587995	5.196510	4.407295
O	5.486670	1.641610	-0.796225	O	9.349784	4.482848	2.409638
H	5.356413	0.701335	-0.983926	H	10.142191	4.019703	2.718054
H	4.872069	2.095742	-1.389362	H	9.567334	5.421665	2.317599

Cp-S				$TS2_N$			
Ti	5.965185	2.048140	0.812620	Ti	7.685211	3.483823	1.909260
N	4.607233	2.903068	5.114244	N	4.466753	2.701113	4.164869
N	6.881042	3.505975	6.664569	N	7.286126	3.841081	4.321166
C	8.317464	1.961547	1.143185	C	8.862789	1.539330	2.878536
C	5.197569	4.136396	1.701325	C	6.095267	3.579144	0.122573
C	4.365347	3.814777	0.603153	C	6.954489	4.667685	-0.115346
C	8.054747	1.544830	-0.190272	C	9.143264	1.522259	1.504989
C	7.151675	-0.005576	1.227330	C	6.886059	1.250023	1.764077
C	7.357747	0.321471	-0.129758	C	7.924710	1.391626	0.803641
C	6.485798	4.139055	-0.190463	C	5.860642	5.060162	1.850769
C	7.761817	1.006971	2.015150	C	7.465812	1.343975	3.039748
C	6.508711	4.321773	1.218106	C	5.421538	3.813214	1.346711
C	5.162816	3.835443	-0.564860	C	6.807230	5.583388	0.949182
S	4.813112	1.081891	2.759009	S	3.501611	-0.051407	3.192554
C	3.389606	1.932157	3.322495	C	2.607936	1.335793	3.502968
C	4.503082	3.609764	6.175971	C	4.808160	3.835158	4.646016
N	3.445827	2.673622	4.442400	N	3.149672	2.522527	3.942332
H	2.582300	3.083376	4.802801	H	2.519044	3.291998	4.162391
N	2.201993	1.780146	2.766724	N	1.276852	1.401483	3.353758
C	1.879017	1.021922	1.564474	C	0.439129	0.299545	2.917099
H	1.423524	2.271848	3.194468	H	0.810796	2.277119	3.549708
C	7.903234	3.847985	7.443798	C	8.479168	3.966936	4.930645
C	5.659190	3.955322	7.004815	C	6.188779	4.127929	5.063143
H	4.892952	4.233501	2.728982	H	5.955756	2.728961	-0.522618
H	3.309454	3.600940	0.641248	H	7.627177	4.770805	-0.951675
H	4.826274	3.609232	-1.564311	H	7.344663	6.509693	1.062002
H	7.329675	4.227317	-0.854281	H	5.530611	5.539542	2.757035
H	7.367992	4.582638	1.812988	H	4.679394	3.169429	1.781589
H	6.644696	-0.882935	1.596339	H	5.843047	1.074828	1.562992
H	7.002592	-0.243367	-0.977395	H	7.815773	1.344935	-0.267201
H	8.355401	2.059971	-1.087872	H	10.117165	1.642932	1.060793
H	8.861374	2.843966	1.434810	H	9.587511	1.644838	3.668944
H	7.788514	1.045417	3.091902	H	6.937069	1.276609	3.973521
H	1.923420	1.661653	0.681873	H	0.695924	-0.019113	1.906131
H	0.864873	0.643622	1.673260	H	-0.591927	0.645994	2.928660
H	2.562610	0.186457	1.448647	H	0.535821	-0.556938	3.584708
H	3.538336	3.993016	6.511483	H	4.064747	4.589247	4.911211
C	5.439926	4.752392	8.129713	C	6.298768	4.681770	6.342084
C	6.518845	5.101185	8.931331	C	7.538261	4.845975	6.931146
H	4.438582	5.089847	8.363983	H	5.392462	4.938933	6.873688
C	7.778796	4.640362	8.583182	C	8.655583	4.444110	6.215852
H	6.374798	5.719672	9.807843	H	7.626395	5.255935	7.928664
H	8.652688	4.882590	9.173031	H	9.653955	4.512755	6.624371
H	8.877384	3.473133	7.149611	H	9.347584	3.683581	4.356153
O	4.727304	1.056070	-0.591335	O	9.527127	4.036593	0.852919
H	4.713511	0.086877	-0.594389	H	9.670691	3.704348	-0.045269
H	3.808757	1.344896	-0.701044	H	9.633531	5.000182	0.822970
				O	9.003244	5.623858	2.703139
				H	9.817407	5.490416	3.208236
				H	8.482954	6.258955	3.214263

TS2$_S$				Cp-aq			
Ti	6.381507	2.493574	0.636845	C	8.391486	2.211981	1.871291
N	4.788655	2.511395	4.765916	C	4.827986	4.633077	1.383877
N	7.046921	3.293206	6.283012	C	5.864634	4.274961	0.493662
C	8.332469	2.895389	1.957570	C	7.439053	1.708017	0.943375
C	4.412863	3.820823	1.030192	C	6.951342	0.846430	3.005600
C	4.782803	4.042483	-0.305448	C	6.554104	0.866948	1.645114
C	8.724954	2.840795	0.590820	C	6.800365	5.391547	2.257440
C	7.980443	0.767051	1.197912	C	8.097156	1.659532	3.136124
C	8.547689	1.512668	0.148483	C	5.402420	5.304458	2.481747
C	6.450721	4.859590	1.026883	C	7.085295	4.767255	1.027469
C	7.880978	1.620524	2.330117	H	3.784211	4.386352	1.267841
C	5.461323	4.294228	1.863066	H	5.746702	3.740334	-0.434959
C	6.049156	4.679998	-0.309501	H	8.057131	4.675287	0.572330
S	4.992028	0.865725	2.219324	H	7.524771	5.826154	2.928549
C	3.566494	1.432910	3.027458	H	4.874465	5.667422	3.350530
C	4.660365	3.221855	5.823091	H	6.453705	0.318709	3.804844
N	3.625257	2.193976	4.137195	H	5.694692	0.364849	1.228723
H	2.748496	2.492319	4.566002	H	7.402995	1.929614	-0.110374
N	2.355206	1.051785	2.650205	H	9.208051	2.877588	1.644975
C	2.013473	0.327432	1.431441	H	8.625167	1.868010	4.053368
H	1.578670	1.315141	3.246513	Ti	6.285874	3.081178	2.489263
C	8.048923	3.705071	7.055113	H	6.629173	2.889977	5.177148
C	5.798967	3.651354	6.635642	H	6.099267	4.332336	4.892637
H	3.484039	3.383863	1.351909	H	3.674049	2.879977	3.162079
H	4.215874	3.750225	-1.175825	H	4.277109	1.448319	3.314050
H	6.611838	4.971478	-1.182195	O	4.388961	2.298191	2.861852
H	7.358723	5.338546	1.350608	O	6.555294	3.575622	4.495758
H	5.487315	4.263410	2.939714				
H	7.702218	-0.274735	1.158186				
H	8.750447	1.146357	-0.843416				
H	9.118641	3.659142	0.009356				
H	8.392922	3.752064	2.606974				
H	7.519041	1.344806	3.304436				
H	2.299542	0.892498	0.545195				
H	0.934561	0.196176	1.429941				
H	2.486530	-0.653264	1.410791				
H	3.676894	3.540612	6.172687				
C	5.533290	4.427675	7.765604				
C	6.590986	4.849619	8.559752				
H	4.512310	4.690463	8.010499				
C	7.877735	4.481215	8.199341				
H	6.410397	5.452305	9.440514				
H	8.736814	4.782442	8.783744				
H	9.044832	3.402657	6.750741				
O	6.631548	2.191444	-1.552607				
H	5.786725	2.198593	-2.025891				
H	7.210062	2.821118	-2.007698				
O	4.841191	0.537333	-0.821511				
H	4.447444	0.068646	-0.071824				
H	5.512288	-0.066003	-1.170512				

Table S10: Cartesian coordinates (Å) of **Ti3a^{2+}**, **TS1$_N$***, **TS1$_S$***, **Cp*-N**, **Cp*-S**, **Cp*-N2**, **TS2$_N$***, **TS2$_S$***and **Cp*-aq**:

Ti3a^{2+}				TS1$_N$*			
Ti	0.697152	9.371287	3.615520	Ti	0.603953	9.099849	3.518841
N	2.530241	8.732303	4.653829	N	2.494551	8.313499	4.592902
C	-0.664758	7.728427	4.823355	C	-0.647952	7.796519	5.192559
C	0.930213	10.833405	1.695717	C	1.195620	11.001058	1.997302
C	-1.626115	9.739961	4.251760	C	-1.873788	9.265524	3.915346
C	-0.009163	9.835817	1.320072	C	-0.012180	10.446252	1.516579
C	2.200777	10.205189	1.851364	C	2.220742	10.016621	1.858983
C	-0.244072	8.699595	5.775258	C	-0.484725	9.089648	5.741170
C	-1.532139	8.362558	3.895182	C	-1.515227	7.904095	4.062776
C	0.659627	8.575161	1.326788	C	0.243963	9.103683	1.106139
C	-0.851008	9.938257	5.427375	C	-1.195185	10.015205	4.914334
C	2.028991	8.816504	1.634372	C	1.639870	8.862435	1.288111
S	1.979688	11.222351	4.872405	S	1.971536	10.796440	4.937971
C	2.984564	9.878586	5.212498	C	2.955456	9.436945	5.204313
N	3.255561	7.642977	4.928431	N	3.286638	7.256438	4.830238
C	2.900256	6.504771	4.447961	C	2.999937	6.072280	4.421696
N	4.085173	9.942613	5.948306	N	4.061468	9.468455	5.939075
C	4.597243	11.156656	6.563027	C	4.584910	10.657795	6.589250
H	4.588079	9.075987	6.081900	H	4.572591	8.600512	6.016184
N	4.781450	5.457391	5.549545	N	5.033755	5.259082	5.453614
C	5.574657	4.430132	5.891526	C	5.929758	4.325286	5.810858
C	3.691269	5.334129	4.754532	C	3.910988	4.994804	4.743424
H	5.001157	6.387204	5.900455	H	5.189870	6.226896	5.726453
C	3.526033	10.879646	1.986957	C	3.688194	10.226409	2.046317
C	0.657400	12.304625	1.730962	C	1.382269	12.453229	2.302662
C	-1.326902	10.138786	0.681573	C	-1.196007	11.279666	1.140783
C	0.135392	7.273520	0.803010	C	-0.697064	8.268357	0.294228
C	3.141198	7.829394	1.478312	C	2.440980	7.707007	0.773023
H	3.434443	11.912276	2.315199	H	3.901826	11.014916	2.765046
H	4.022974	10.884365	1.010490	H	4.140625	10.520693	1.092848
H	4.188936	10.355927	2.677011	H	4.192932	9.316974	2.372763
H	0.667883	12.715027	0.715673	H	1.318994	13.024798	1.370024
H	1.407943	12.843541	2.307689	H	2.351250	12.661120	2.750326
H	-0.322232	12.524962	2.157310	H	0.606103	12.841180	2.964270
H	-1.807299	11.014003	1.111983	H	-1.507898	11.955823	1.935958
H	-2.022651	9.305213	0.710594	H	-2.048303	10.681936	0.832679
H	-1.144707	10.364882	-0.375544	H	-0.912010	11.905165	0.286664
H	-0.932525	7.317387	0.605926	H	-1.724618	8.341490	0.646670
H	0.323755	6.442030	1.484437	H	-0.415304	7.217277	0.282119
H	0.635093	7.032619	-0.140789	H	-0.688851	8.614360	-0.745350
H	3.968549	8.022484	2.159851	H	3.025120	7.211166	1.551555
H	3.539317	7.906133	0.460049	H	3.159345	8.069282	0.031371
H	2.806710	6.802853	1.616245	H	1.819329	6.966996	0.269916
C	0.518511	8.433871	7.033025	C	0.106543	9.363319	7.086043
C	-0.910187	11.166561	6.277611	C	-1.415831	11.466577	5.205490
C	-2.599350	10.746790	3.720402	C	-3.056930	9.728363	3.128653
C	-2.447905	7.613873	2.980595	C	-2.245616	6.774666	3.403312
C	-0.449370	6.248975	4.898229	C	-0.203046	6.529037	5.850727

H	1.130659	7.536847	6.957663	H	1.072548	8.877256	7.230314
H	-0.180527	8.284770	7.862719	H	-0.567754	8.964218	7.852215
H	1.162696	9.269488	7.307334	H	0.227141	10.427015	7.274690
H	-0.080259	11.223078	6.978277	H	-0.570676	11.912270	5.729354
H	-1.837493	11.148784	6.861578	H	-2.294811	11.591174	5.847325
H	-0.922969	12.080389	5.682927	H	-1.595936	12.041174	4.297009
H	-2.126365	11.701166	3.482651	H	-3.085426	10.807047	3.006614
H	-3.360505	10.949156	4.481017	H	-3.957526	9.441719	3.684769
H	-3.112483	10.388160	2.832426	H	-3.129004	9.258954	2.149514
H	-2.850621	8.229354	2.181642	H	-2.304538	6.876975	2.318070
H	-3.299927	7.254561	3.569358	H	-3.278425	6.758900	3.767500
H	-1.973875	6.737993	2.542727	H	-1.811847	5.805586	3.644794
H	-0.237698	5.812182	3.921472	H	-0.067761	5.716824	5.140141
H	-1.361435	5.768735	5.269357	H	-0.962639	6.207812	6.572435
H	0.358123	5.983963	5.577739	H	0.726230	6.664069	6.402873
H	4.840241	11.907157	5.809621	H	4.840335	11.428781	5.860404
H	5.502912	10.899063	7.106096	H	5.485216	10.374581	7.128987
H	3.875234	11.578960	7.263342	H	3.865751	11.069355	7.298932
H	2.035259	6.367416	3.809579	H	2.107543	5.818168	3.868568
C	3.384849	4.066766	4.267125	C	3.683850	3.674248	4.361859
C	4.185086	2.987148	4.603105	C	4.590452	2.689117	4.714406
H	2.519371	3.948271	3.631050	H	2.794167	3.441904	3.793999
C	5.296794	3.165484	5.426495	C	5.731188	3.013077	5.450580
H	3.945308	2.002692	4.223804	H	4.411735	1.663969	4.418096
H	5.936687	2.341040	5.703154	H	6.452717	2.263344	5.738531
H	6.411668	4.665124	6.531877	H	6.781178	4.671497	6.377764
				O	0.567944	6.322305	2.653811
				H	1.197817	6.159624	1.940404
				H	-0.293864	6.167234	2.248654

**TS1$_S$***				**Cp*-N**			
Ti	0.603826	9.691950	3.643028	Ti	0.565881	9.020774	3.473187
N	2.520850	9.013220	4.719972	N	2.493485	8.210086	4.650441
C	-0.637568	7.758100	4.533507	C	-0.740794	7.756987	5.168807
C	1.387889	10.861514	1.614536	C	1.178049	11.009714	2.026386
C	-1.722164	9.787738	4.578389	C	-1.911182	9.243881	3.860734
C	0.151645	10.218767	1.299823	C	-0.041534	10.507603	1.519523
C	2.354161	9.858785	1.869672	C	2.186853	10.014802	1.825712
C	-0.170973	8.484721	5.667002	C	-0.554720	9.046303	5.710023
C	-1.617204	8.564288	3.880059	C	-1.575043	7.878376	4.016257
C	0.386179	8.814781	1.305471	C	0.184819	9.182694	1.045452
C	-0.807405	9.755827	5.671004	C	-1.231026	9.983563	4.867347
C	1.733554	8.590822	1.679407	C	1.579556	8.913569	1.189743
S	1.943844	11.477178	4.916456	S	1.975736	10.693830	4.945963
C	2.975752	10.165121	5.265492	C	2.972981	9.349322	5.215753
N	3.297754	7.959615	4.987590	N	3.307461	7.166973	4.870387
C	2.970565	6.778955	4.599787	C	2.985752	5.948066	4.607829
N	4.083444	10.249323	5.989246	N	4.098877	9.398244	5.921432
C	4.604111	11.481651	6.558397	C	4.651019	10.604622	6.512543
H	4.607810	9.393855	6.110237	H	4.616199	8.533609	5.995088
N	4.952495	5.885746	5.659547	N	5.170804	5.238486	5.376212

C	5.801889	4.915083	6.032458	C	6.123933	4.341784	5.678548
C	3.828512	5.666063	4.935652	C	3.949601	4.907459	4.891427
H	5.148596	6.846075	5.933008	H	5.358278	6.228591	5.517733
C	3.812164	10.113179	2.069241	C	3.659815	10.188187	2.015147
C	1.715466	12.312877	1.449434	C	1.399352	12.444816	2.387327
C	-1.070965	10.882058	0.745035	C	-1.203672	11.387571	1.183050
C	-0.467942	7.777315	0.650758	C	-0.768971	8.360647	0.234718
C	2.441159	7.284157	1.502477	C	2.324485	7.760509	0.594030
H	3.991599	10.961812	2.730562	H	3.890035	10.931057	2.776220
H	4.277134	10.355079	1.107162	H	4.116121	10.525405	1.077899
H	4.329504	9.244563	2.472449	H	4.148960	9.253234	2.290239
H	2.389311	12.433449	0.595299	H	1.346662	13.054954	1.478451
H	2.232306	12.730526	2.315874	H	2.374465	12.612308	2.838521
H	0.832865	12.915840	1.237604	H	0.636224	12.826159	3.067678
H	-1.170023	11.910779	1.083076	H	-1.500049	12.036707	2.005944
H	-1.983733	10.350154	1.009264	H	-2.071170	10.828178	0.847027
H	-1.015474	10.899086	-0.349111	H	-0.901090	12.043369	0.358390
H	-1.487628	8.115574	0.492260	H	-1.807266	8.593073	0.462904
H	-0.489291	6.835525	1.197115	H	-0.622248	7.289665	0.380283
H	-0.040624	7.560771	-0.335325	H	-0.618809	8.557806	-0.832612
H	3.403403	7.258512	2.008586	H	3.104347	7.371454	1.255075
H	2.633293	7.133339	0.433593	H	2.835085	8.090562	-0.316493
H	1.845509	6.435805	1.837562	H	1.664300	6.943178	0.302572
C	0.619040	7.954095	6.819787	C	0.024243	9.316230	7.061224
C	-0.762722	10.729236	6.806760	C	-1.436115	11.435133	5.169544
C	-2.834947	10.767259	4.377089	C	-3.079657	9.721653	3.059642
C	-2.653876	8.071307	2.921626	C	-2.235262	6.749913	3.287125
C	-0.446296	6.294882	4.264171	C	-0.328384	6.480066	5.827442
H	1.140433	7.030527	6.581483	H	0.973675	8.803676	7.222826
H	-0.067612	7.737143	7.645315	H	-0.671942	8.945744	7.822309
H	1.345579	8.674481	7.196063	H	0.174972	10.377581	7.240759
H	0.198791	10.714989	7.316784	H	-0.578485	11.871602	5.681187
H	-1.530301	10.466482	7.543538	H	-2.301965	11.563267	5.828627
H	-0.960244	11.752987	6.489709	H	-1.627826	12.017433	4.269193
H	-2.693569	11.683698	4.946816	H	-3.106384	10.802250	2.958152
H	-3.768082	10.311682	4.727338	H	-3.990768	9.424282	3.592215
H	-2.981656	11.027832	3.329388	H	-3.133367	9.271820	2.070130
H	-2.923255	8.812627	2.171257	H	-2.386331	6.970731	2.230413
H	-3.565206	7.850969	3.490037	H	-3.224607	6.562013	3.718528
H	-2.357829	7.155301	2.420106	H	-1.679845	5.815367	3.374747
H	-0.054694	6.087961	3.267263	H	-0.206846	5.666893	5.114242
H	-1.410042	5.782143	4.338431	H	-1.100106	6.171636	6.541842
H	0.220269	5.836312	4.992070	H	0.600339	6.591911	6.386049
H	4.864914	12.199618	5.779094	H	4.909025	11.341235	5.749396
H	5.500506	11.236428	7.122476	H	5.553575	10.329764	7.053182
H	3.880132	11.940366	7.232793	H	3.948158	11.057620	7.213112
H	2.072987	6.558152	4.041412	H	2.031140	5.633047	4.210608
C	3.549006	4.354986	4.556212	C	3.679773	3.554223	4.693766
C	4.406969	3.333161	4.925465	C	4.641838	2.606901	4.997025
H	2.657519	4.158982	3.977854	H	2.712527	3.267700	4.305955
C	5.550787	3.611065	5.675507	C	5.884628	3.000356	5.497409
H	4.187523	2.315338	4.631346	H	4.428048	1.557110	4.845436

H	6.234597	2.831915	5.977135	H	6.652430	2.281207	5.740025
H	6.659918	5.226494	6.609458	H	7.052999	4.740877	6.057061
O	-0.501008	12.191587	3.520481	O	0.755219	6.875567	2.902835
H	0.070401	12.874956	3.146473	H	1.520562	6.660845	2.351778
H	-0.766655	12.540139	4.381440	H	-0.002631	6.513487	2.422151

**Cp*-S**				**Cp*-N2**			
Ti	0.151420	10.010669	3.444700	Ti	0.286602	9.284486	3.408470
N	3.081447	8.123558	4.854703	N	3.228688	7.713109	5.058083
C	-0.994218	8.055980	4.323091	C	-0.830580	8.611144	5.486536
C	1.105381	11.068313	1.477135	C	1.067399	11.203011	2.105923
C	-2.080997	10.085274	4.416617	C	-2.134548	9.070832	3.646806
C	-0.155271	10.508586	1.106877	C	-0.133384	10.782822	1.479416
C	1.989741	10.005420	1.790125	C	2.046750	10.177049	1.908720
C	-0.514218	8.762396	5.460296	C	-1.000261	10.029868	5.400137
C	-1.996481	8.864282	3.700868	C	-1.548881	8.026484	4.418354
C	-0.027000	9.094417	1.153843	C	0.064510	9.460951	0.985386
C	-1.166373	10.024365	5.505472	C	-1.811681	10.311181	4.278748
C	1.276491	8.783193	1.634147	C	1.423661	9.113029	1.221719
S	2.152038	10.618892	4.944344	S	2.142280	10.147773	5.134383
C	3.283073	9.370758	5.325576	C	3.362157	9.004820	5.464750
N	4.011193	7.255723	5.261627	N	4.191032	6.817975	5.314202
C	3.863329	6.021573	4.894496	C	4.036448	5.621204	4.879967
N	4.330205	9.684612	6.097609	N	4.448544	9.329361	6.150149
C	4.618762	10.983786	6.676544	C	4.746168	10.654706	6.669879
H	4.949830	8.914721	6.309980	H	5.101632	8.581443	6.344719
N	5.877605	5.417261	6.079310	N	6.151678	4.980249	5.865660
C	6.818076	4.570582	6.533679	C	7.151822	4.136031	6.154823
C	4.821052	5.037484	5.316552	C	5.064157	4.631680	5.139247
H	5.944174	6.404759	6.313818	H	6.203340	5.937552	6.207622
C	3.466528	10.137948	1.964757	C	3.518223	10.287985	2.154276
C	1.499678	12.506802	1.357846	C	1.320294	12.568365	2.668496
C	-1.325748	11.276573	0.577846	C	-1.251626	11.693908	1.085215
C	-0.913285	8.110021	0.462727	C	-0.868334	8.681014	0.110833
C	1.883568	7.416167	1.639956	C	2.105074	7.893414	0.691495
H	3.742632	11.090263	2.413992	H	3.758490	11.059240	2.882384
H	3.942325	10.088708	0.978499	H	4.020082	10.552987	1.216959
H	3.883447	9.335195	2.569136	H	3.955039	9.348015	2.494415
H	2.056822	12.659157	0.428320	H	1.572931	13.270930	1.866970
H	2.155336	12.830213	2.169608	H	2.151394	12.568856	3.372663
H	0.635160	13.171289	1.309360	H	0.443324	12.964820	3.181645
H	-1.458571	12.226341	1.096260	H	-1.335440	12.554887	1.742815
H	-2.252304	10.711109	0.660678	H	-2.217347	11.197319	1.034448
H	-1.174912	11.506071	-0.482367	H	-1.037306	12.075788	0.080166
H	-1.913324	8.495687	0.287929	H	-1.869604	9.104652	0.105313
H	-0.984911	7.158215	0.984187	H	-0.942697	7.634906	0.413276
H	-0.471041	7.901184	-0.518347	H	-0.501613	8.688980	-0.921081
H	2.713710	7.344823	2.339333	H	2.958667	7.593092	1.303482
H	2.269505	7.186182	0.640186	H	2.495705	8.089766	-0.312433
H	1.156184	6.645468	1.888874	H	1.422155	7.046149	0.602622
C	0.307023	8.184634	6.564574	C	-0.637865	11.047244	6.436539

C	-1.037409	11.012780	6.620193	C	-2.456286	11.642167	4.046839
C	-3.089699	11.156543	4.147591	C	-3.188803	8.846106	2.607399
C	-3.049242	8.404584	2.743226	C	-1.771276	6.566598	4.192667
C	-0.722001	6.611712	4.041595	C	-0.160374	7.867942	6.598557
H	0.965722	7.392683	6.216898	H	0.089480	10.670454	7.152058
H	-0.368742	7.753718	7.312488	H	-1.537944	11.326802	6.995657
H	0.909856	8.939068	7.068363	H	-0.241343	11.964317	5.997295
H	-0.000016	11.150529	6.926385	H	-1.734962	12.459751	4.015283
H	-1.588534	10.652785	7.495056	H	-3.137325	11.854036	4.878561
H	-1.459386	11.986744	6.367771	H	-3.043404	11.665457	3.133325
H	-2.828691	12.102461	4.620407	H	-3.329455	9.706302	1.957782
H	-4.063103	10.853355	4.547762	H	-4.147038	8.655410	3.104105
H	-3.220692	11.331585	3.079481	H	-2.973835	7.977105	1.987205
H	-3.283328	9.148546	1.983129	H	-1.794004	6.315363	3.131678
H	-3.969784	8.230592	3.311424	H	-2.736044	6.267898	4.617073
H	-2.790661	7.470554	2.254459	H	-1.003622	5.959503	4.669702
H	-0.950417	6.339938	3.013367	H	0.276868	6.930124	6.254604
H	-1.348581	5.989054	4.689665	H	-0.888154	7.617344	7.377700
H	0.314601	6.343268	4.243814	H	0.626092	8.458508	7.066801
H	4.749797	11.745201	5.906130	H	4.749163	11.394179	5.869451
H	5.543886	10.897924	7.242419	H	5.733681	10.616295	7.122122
H	3.824894	11.307413	7.352231	H	4.020546	10.955002	7.426693
H	3.028054	5.688204	4.284025	H	3.166638	5.298942	4.312377
C	4.721831	3.682598	4.988070	C	4.981771	3.328493	4.668286
C	5.674211	2.790422	5.441073	C	6.002603	2.431577	4.949805
H	3.889624	3.356076	4.380516	H	4.119019	3.035524	4.087093
C	6.742198	3.234237	6.228264	C	7.102261	2.835723	5.701787
H	5.592113	1.742208	5.185471	H	5.940827	1.415755	4.583016
H	7.497722	2.555505	6.594481	H	7.909338	2.157867	5.935956
H	7.603429	5.007645	7.132262	H	7.962664	4.537665	6.744117
O	-0.149629	12.069624	3.752634	O	0.959616	7.497047	3.448268
H	0.285625	12.774815	3.251460	H	2.382425	7.463338	4.518979
H	-0.523758	12.473650	4.547966	H	1.516580	7.212168	2.714368

**$TS2_N$***				**$TS2_S$***			
Ti	0.084771	9.072075	2.942643	Ti	-0.105653	10.259857	3.315371
N	3.421043	7.977407	5.290503	N	3.124158	8.201734	4.851882
C	-1.269720	7.488322	4.197641	C	-1.049435	8.207969	4.253588
C	1.031871	10.985070	1.785457	C	1.002671	11.100442	1.312637
C	-2.241172	9.495434	3.614144	C	-2.282075	10.130770	4.552747
C	-0.308455	10.890468	1.336807	C	-0.197781	10.490581	0.835891
C	1.732806	9.819815	1.355870	C	1.855368	10.066802	1.775672
C	-0.806021	8.470989	5.100790	C	-0.537580	8.894399	5.382099
C	-2.125431	8.121160	3.249236	C	-2.131834	8.986441	3.723948
C	-0.458531	9.646650	0.655772	C	-0.115637	9.103098	1.080810
C	-1.383987	9.727887	4.719533	C	-1.262707	10.114070	5.531252
C	0.810076	8.992821	0.658578	C	1.141508	8.836649	1.702432
S	2.605685	10.489111	5.195485	S	2.073654	10.670854	4.861368
C	3.629811	9.260742	5.735976	C	3.250917	9.476122	5.277887
N	4.229846	6.983872	5.663699	N	4.110628	7.403973	5.265137
C	4.003417	5.800074	5.222426	C	4.010016	6.147825	4.954794

N	4.646066	9.443082	6.570860	N	4.280129	9.860080	6.046085
C	5.028894	10.723397	7.141087	C	4.521656	11.186751	6.580486
H	5.197597	8.629490	6.808411	H	4.933163	9.124243	6.278279
N	5.939908	4.982599	6.420494	N	6.110037	5.674737	6.050520
C	6.813222	4.050915	6.829924	C	7.105264	4.882022	6.487087
C	4.886791	4.719301	5.611688	C	5.027133	5.223981	5.366958
H	6.068517	5.943799	6.728942	H	6.154903	6.673501	6.236380
C	3.205920	9.591805	1.490871	C	3.330441	10.197091	1.964024
C	1.619193	12.209490	2.413153	C	1.386901	12.536582	1.141994
C	-1.249261	12.053394	1.317495	C	-1.253585	11.148183	0.007175
C	-1.619181	9.234707	-0.192315	C	-0.987955	8.081071	0.425544
C	1.116210	7.716003	-0.060202	C	1.767228	7.483207	1.825327
H	3.579608	9.904749	2.465963	H	3.617252	11.167473	2.364105
H	3.741848	10.168932	0.730605	H	3.805740	10.094105	0.980696
H	3.478722	8.546505	1.338447	H	3.737064	9.420143	2.606188
H	1.650505	13.016715	1.673177	H	1.843335	12.695537	0.159123
H	2.633537	12.040965	2.766286	H	2.118649	12.849209	1.888245
H	1.018238	12.569744	3.250105	H	0.524598	13.199738	1.207462
H	-1.334256	12.542739	2.288000	H	-1.220934	12.232723	0.084742
H	-2.244045	11.779291	0.978584	H	-2.258999	10.809228	0.264485
H	-0.860153	12.801808	0.618523	H	-1.094461	10.889407	-1.045128
H	-2.560254	9.651369	0.159112	H	-1.991891	8.453039	0.235364
H	-1.720840	8.152431	-0.250874	H	-1.059288	7.152815	0.985855
H	-1.461134	9.599041	-1.213411	H	-0.546148	7.833729	-0.546992
H	2.035272	7.254762	0.297645	H	2.378872	7.397957	2.720996
H	1.246223	7.914052	-1.129236	H	2.416550	7.310285	0.959050
H	0.308591	6.989926	0.036492	H	1.023578	6.690183	1.833182
C	-0.017534	8.206144	6.343197	C	0.323151	8.296803	6.443988
C	-1.251223	11.016420	5.471203	C	-1.126914	11.063059	6.678580
C	-3.303145	10.428549	3.130119	C	-3.444836	11.067540	4.526775
C	-2.974293	7.384426	2.260011	C	-3.180308	8.534588	2.754500
C	-1.018946	6.019721	4.278967	C	-0.774183	6.767345	3.958851
H	0.617448	7.326565	6.240672	H	1.016182	7.557661	6.052622
H	-0.704358	8.013772	7.174781	H	-0.331716	7.794570	7.166576
H	0.608212	9.051612	6.622665	H	0.890969	9.048658	6.989065
H	-0.278183	11.105120	5.954012	H	-0.085510	11.196427	6.973859
H	-2.011404	11.074615	6.257867	H	-1.660448	10.672700	7.551973
H	-1.389421	11.880911	4.821466	H	-1.549681	12.041038	6.451082
H	-3.055459	11.470941	3.315034	H	-3.186746	12.072981	4.860345
H	-4.224569	10.212225	3.683176	H	-4.211003	10.691949	5.214144
H	-3.529175	10.303754	2.073602	H	-3.911367	11.131871	3.543749
H	-3.512754	8.060548	1.600845	H	-3.404300	9.288512	1.998166
H	-3.716436	6.783185	2.795763	H	-4.111217	8.342530	3.298397
H	-2.394232	6.695448	1.644690	H	-2.902797	7.613608	2.250966
H	-1.064274	5.547680	3.297479	H	-1.023727	6.488656	2.938739
H	-1.787155	5.549294	4.902374	H	-1.389870	6.148179	4.621454
H	-0.052824	5.792674	4.726571	H	0.264434	6.498684	4.143849
H	5.341779	11.424331	6.366100	H	4.610717	11.928811	5.785694
H	5.862059	10.547526	7.817393	H	5.456858	11.155605	7.135827
H	4.203374	11.164896	7.699486	H	3.724177	11.500149	7.256871
H	3.177928	5.556573	4.558108	H	3.165695	5.751936	4.396793
C	4.704362	3.410234	5.183526	C	4.961029	3.851613	5.105007

C	5.591664	2.424654	5.589855	C	5.968467	3.014904	5.541980
H	3.869309	3.182920	4.536516	H	4.108966	3.468791	4.561207
C	6.660899	2.744045	6.423318	C	7.062239	3.531520	6.246223
H	5.450756	1.405288	5.256091	H	5.910815	1.953686	5.338515
H	7.366398	1.996362	6.753415	H	7.861914	2.896952	6.597593
H	7.610446	4.392095	7.473443	H	7.906275	5.373290	7.018999
O	1.306704	7.631508	3.204215	O	-1.410420	11.948140	2.680106
H	2.633051	7.808649	4.653910	H	-1.524553	12.570813	3.413047
H	2.067691	7.645003	2.609688	H	-2.281964	11.764234	2.305743
O	1.588612	4.700815	2.221441	O	0.222277	12.695112	4.357254
H	0.700112	4.513545	1.891076	H	0.607953	12.749131	5.242700
H	1.528234	5.603513	2.573410	H	0.814686	13.211258	3.791101

**Cp*-aq**			
C	8.526641	2.238765	2.284200
C	4.605043	4.644205	1.733458
C	5.445406	4.300338	0.633795
C	7.812657	1.739655	1.151325
C	6.924773	0.766132	3.040396
C	6.805427	0.856379	1.621295
C	6.736428	5.358923	2.220281
C	7.990728	1.613287	3.442256
C	5.397803	5.293648	2.708846
C	6.749327	4.802936	0.910693
Ti	6.233712	3.021146	2.523707
H	6.114549	2.626378	5.221196
H	5.555068	4.050559	4.968323
H	3.565668	2.526644	2.872193
H	4.303128	1.178754	3.094856
O	4.382461	2.045893	2.672059
O	6.134193	3.367673	4.597950
C	8.239305	1.823066	-0.279178
H	7.713387	4.404621	-0.976796
H	8.702602	0.868208	-0.550420
H	7.404879	1.974344	-0.961678
C	9.794979	3.032101	2.282727
H	10.644607	2.352711	2.409984
H	9.949489	3.572209	1.352712
H	9.828217	3.744550	3.106740
C	8.572235	1.711400	4.815311
H	9.446452	1.055355	4.882850
H	8.908839	2.721421	5.048391
H	7.874409	1.387747	5.587937
C	6.183064	-0.171487	3.941736
H	6.834570	-1.005539	4.219556
H	5.865778	0.305805	4.870730
H	5.309101	-0.609598	3.457863
C	5.902386	0.045205	0.747797
H	5.617253	0.588907	-0.152680
H	6.419179	-0.866153	0.428992
H	4.994193	-0.259769	1.266090

C	3.118534	4.500881	1.763727
H	2.657414	5.408318	1.361234
H	2.772131	3.669174	1.149772
H	2.730077	4.382505	2.778117
C	4.887861	5.953299	3.948595
H	4.507641	6.949477	3.701725
H	4.055931	5.407803	4.400040
H	5.671425	6.096282	4.694546
C	7.863287	6.083198	2.886170
H	7.737579	7.163623	2.759017
H	7.895585	5.887495	3.958770
H	8.826365	5.812173	2.458765
C	4.957458	3.710350	-0.651521
H	4.298159	2.858100	-0.480520
H	4.381052	4.456239	-1.208352
H	5.776196	3.385229	-1.289133
C	7.826772	5.032613	-0.098317
H	8.978228	2.600356	-0.448534
H	7.750093	6.073503	-0.431821
H	8.827711	4.902541	0.304993

References

[1] L. Krause, R. Herbst-Irmer, G. M. Sheldrick, D. Stalke, Comparison of silver and molybdenum microfocus X-ray sources for single-crystal structure determination, *J. Appl. Cryst.* **2015**, *48*, 3-10.

[2] G. Sheldrick, A short history of SHELX, *Acta Cryst. A* **2008**, *64*, 112-122.

[3] G. Sheldrick, Crystal structure refinement with SHELXL, *Acta Cryst. C* **2015**, *71*, 3-8.

[4] O. V. Dolomanov, L. J. Bourhis, R. J. Gildea, J. A. K. Howard, H. Puschmann, OLEX2: a complete structure solution, refinement and analysis program, *J. Appl. Cryst.* **2009**, *42*, 339-341.

[5] F. Weigend, R. Ahlrichs, Balanced basis sets of split valence, triple zeta valence and quadruple zeta valence quality for H to Rn: Design and assessment of accuracy, *Phys. Chem. Chem. Phys.* **2005**, *7*, 3297-3305.

[6] A. Marenich, C. Cramer, D. Truhlar, Universal Solvation Model Based on Solute Electron Density and on a Continuum Model of the Solvent Defined by the Bulk Dielectric Constant and Atomic Surface Tensions, *J. Phys. Chem. B* **2009**, 113, *18*, 6378–6396.
